# Is Incentive Spirometry Superior to Standard Care in Postoperative Cardiac Surgery on Clinical Outcomes and Length of Hospital and Intensive Care Unit Stay? A Systematic Review with Meta-Analysis

**DOI:** 10.21470/1678-9741-2022-0319

**Published:** 2024-04-15

**Authors:** Hiago Vinicius Costa Silva, Adriana Claudia Lunardi, Ana Carolina Pereira Nunes Pinto, Juliana Ribeiro Fonseca Franco de Macedo, Elinaldo da Conceição dos Santos

**Affiliations:** 1 Department of Biological and Health Sciences, Universidade Federal do Amapá, Macapá, Amapá, Brazil; 2 Programa de Pós-Graduação Stricto Sensu em Fisioterapia, Universidade Cidade de São Paulo, São Paulo, São Paulo, Brazil; 3 Department of Physical Therapy, Escola de Medicina, Universidade de São Paulo, São Paulo, São Paulo, Brazil; 4 Department of Physical Therapy, University of Pittsburgh, Pennsylvania, United States of America; 5 Department of Health Sciences, Catholic University of Louvain, Brussels, Belgium

**Keywords:** Cardiac Surgical Procedures, Postoperative Care, Noninvasive Ventilation, Systematic Review

## Abstract

**Introduction:**

Cardiac surgery is a frequent surgical procedure and may present a high risk of complications. Among the prophylactic strategies studied to decrease the rates of negative outcomes, respiratory care seems to reduce pulmonary complications. Incentive spirometry (IS) is a low-cost, respiratory exercise technique, used for the prevention and treatment of postoperative pulmonary complications (PPC). The aim of this review was to evaluate whether IS is superior to respiratory care, mobilization exercises, and noninvasive ventilation on PPC, and clinical outcomes.

**Methods:**

Systematic review. Medical Literature Analysis and Retrieval System Online (or MEDLINE®), Embase®, Cochrane Central Register of Controlled Trials (or CENTRAL), Physiotherapy Evidence Database (or PEDro), Cumulative Index of Nursing and Allied Health (or CINAHL®), Latin American and Caribbean Health Sciences Literature (or LILACS), Scientific Electronic Library Online (or SciELO), Allied, Scopus®, and OpenGrey databases, clinical trial registration sites, conferences, congresses, and symposiums were searched.

**Results:**

Twenty-one randomized trials and one quasi-randomized trial (1,677 participants) were included. For partial pressure of oxygen (PaO2), IS was inferior to respiratory care (mean difference [MD] -4.48; 95% confidence interval [CI] -8.32 to -0.63). Flow-oriented IS was inferior to respiratory care on PaO2 (MD -4.53; 95% CI -8.88 to -0.18). However, compared to respiratory care, flow-oriented IS was superior on recovery vital capacity.

**Conclusions:**

This meta-analysis revealed that IS was not superior to standard respiratory care for PPCs and clinical outcomes, therefore its use should not be widely recommended until further studies with high quality be performed to ensure this clinical guidance.

## INTRODUCTION

**Table:** 

Abbreviations, Acronyms & Symbols				
CABG	= Coronary artery bypass grafting		MD	= Mean differences
CENTRAL	= Cochrane Central Register of Controlled Trials		MIP	= Maximal inspiratory pressure
CG	= Control group		NIV	= Noninvasive ventilation
CI	= Confidence interval		NR	= Not registered
CINAHL®	= Cumulative Index of Nursing and Allied Health		PaO_2_	= Partial pressure of oxygen
CPAP	= Continuous positive airway pressure		PEDro	= Physiotherapy Evidence Database
ECC	= Extracorporeal circulation		PEF	= Peak of expiratory flow
FEF	= Forced expiratory flow		PO	= Postoperative
FEV_1_	= Forced expiratory volume in one second		PPC	= Postoperative pulmonary complications
FVC	= Forced vital capacity		RCT	= Randomized controlled trial
GRADE	= Classification of Recommendations, Assessment, Development and Evaluation		RR SciELO	= Risk ratios = Scientific Electronic Library Online
ICU	= Intensive care unit		SD	= Standard deviation
IPPB	= Intermittent positive pressure breathing		SMD	= Standardized mean differences
IS	= Incentive spirometry		SO_2_	= Oxygen saturation
ISG	= Incentive spirometry group		VC	= Vital capacity
LILACS	= Latin American and Caribbean Health Sciences Literature		VR	= Valve replacement
LOS	= Length of stay			

Cardiac surgery is a frequent surgical procedure. Each year, Australian hospitals perform > 12,000 cardiac surgeries, and a single Brazilian hospital has already performed > 2,900 of these procedures^[1,2]^. In the United States of America, the cost of cardiac surgery is approximately 1% to 2% of the health budget^[3]^. The majority of patients undergo coronary artery bypass grafting (CABG), and 74.6% of surgeries are scheduled^[4]^. Complex cardiac surgery and prolonged hospital length of stay (LOS) may present a high risk of complications and mortality; postoperative mortality has been documented at 4% (valve operations) within the first seven days and 6.4% (overall mortality) within the first postoperative month^[4]^.

Approximately 10.2% to 27.3% of CABG patients present at least one complication, 70.6% after valve surgery, and 84.2% after combined surgery (CABG + valve surgery)^[5,6]^. Regarding the complications, 2.2% are major adverse cardiovascular events^[7]^, 7.5% are reintubated during the intensive care unit (ICU) stay, which increases the rate of complications^[8]^, 23.2% remain hospitalized in an ICU for more than two days after surgery, and 59.7% remain hospitalized for more than seven days^[6]^. It seems that when the complication rate increases, hospital LOS and mortality also increase (12% in the ICU and 15.1% in the 30-day period), mainly in older adults^[5,9]^.

Among the prophylactic strategies to decrease these rates of negative outcomes, respiratory care seems to reduce pulmonary complications and minimize postoperative pulmonary dysfunction^[10]^. As one of the respiratory care techniques, incentive spirometry (IS) is a low-cost, widespread, respiratory exercise technique, used for the prevention and treatment of postoperative pulmonary complications (PPC) in patients undergoing cardiac surgery^[11]^. IS is a device that provides visual feedback when the patient inhales at a predetermined flow or volume. The patient is required to place the lips firmly around the mouthpiece and to inhale slowly to raise the ball (flow-oriented) or piston/plate (volume-oriented) in the chamber toward the defined target^[12]^.

It has been suggested that patients undergoing cardiac surgery who are more adherent to IS therapy may benefit from a reduced LOS and a reduction in the mortality rate^[13]^. On the other hand, scientific evidence has suggested that IS does not improve clinical outcomes in different surgical patients^[14]^. In order to strengthen the scientific findings, our systematic review, performed with strict methodological criteria, is intended to clarify these specific gaps, exclusively in patients undergoing cardiac surgery and assist clinicians in decision making. Our aim was to assess whether IS is superior to respiratory care, mobilization exercises, and noninvasive ventilation (NIV) on PPC, adverse events, mortality, hospital and/or ICU LOS, lung function, oxygenation, and maximal inspiratory pressure (MIP) in patients undergoing cardiac surgery.

## METHODS

### Design

We conducted a systematic review following the reporting recommendations proposed by the Preferred Reporting Items for Systematic Reviews and Meta-Analysis (or PRISMA)^[15]^. The protocol was registered in the International Prospective Register of Systematic Reviews (or PROSPERO) (#CRD42020161009), is available online at https://www.crd.york.ac.uk/prospero/export_record_pdf.php), and was previously published^[16]^.

### Eligibility Criteria

#### Types of Studies, Participants, and Interventions

We searched for randomized and quasi-randomized controlled trials published in any year, in any language. The studies included in this review were required to have enrolled patients aged 18 years or older, who were breathing spontaneously, undergoing cardiac surgeries, and which evaluated the effects of postoperative flow or volume-oriented IS on our pre-defined clinical outcomes. The treatment comparison was made with standard care, such as respiratory care (maximal inspiratory breathing exercises, coughing and deep breathing, supported/assisted coughing, huffing technique, diaphragmatic breathing, fractional inspiration, active cycle of breathing, and autogenic drainage), NIV, and other therapies (mobilization exercise, blow bottles, and verbal encouragement). The mobilization exercises considered in this review were early mobilization programs, active/passive exercises of upper/lower limbs, and physical therapy.

The controlled trials had to have evaluated at least one of the following outcomes:

#### Primary Outcomes

- PPC: For this systematic review, atelectasis and pneumonia were considered.
- Adverse events: Any reaction, harm, or complication associated with IS reported in the included studies.
- Mortality: All reported deaths were accepted, regardless of cause.

#### Secondary Outcomes

- LOS: The number of days spent in hospital after cardiac surgical procedure.
- Length of ICU stay: The number of days spent in the ICU after cardiac surgical procedure.
- Lung function: Variables evaluated were peak of expiratory flow (PEF), forced expiratory volume in one second (FEV₁), forced vital capacity (FVC), and vital capacity (VC).
- Oxygenation: Arterial partial pressure of oxygen (PaO_2_) and peripheral and central arterial oxygen saturation (SO_2_) were accepted.
- MIP (cmH₂O): MIP measured with digital or analog manovacuometer or manometer was accepted.

### Database and Search Strategy

The search strategy was sensitive (Supplement 1) to capture all potentially qualifying studies through the Medical Literature Analysis and Retrieval System Online (or MEDLINE®), Embase®, Cochrane Central Register of Controlled Trials (or CENTRAL), Physiotherapy Evidence Database (PEDro), Cumulative Index of Nursing and Allied Health (or CINAHL®), Latin American and Caribbean Health Sciences Literature (or LILACS), Scientific Electronic Library Online (or SciELO), and Scopus® databases, as well as in the OpenGrey database, the main clinical trial registration sites, conferences, congresses, and symposiums in the area described in the protocol^[16]^. When necessary, we contacted the authors of the clinical trials to request additional data. The snowball technique, which consists of searching the reference lists of the included studies, was used to optimize the search. The search was performed on July 22 and 24, 2022.

### Study Selection and Data Extraction

Two authors independently selected the studies identified by the search strategy based on eligibility criteria. Duplicate publications were excluded, after which the authors selected the studies by titles and abstracts. Non-randomized trials and studies lacking predefined outcomes were excluded. In some cases, it was necessary to read the full texts. Where reports with the same participants but different outcome measurements or using different time points for the assessments were found, both reports were included. However, the two reports were considered as parts of only one study.

The Rayyan app was used to optimize the process of screening and selecting the studies^[17]^. Disagreements between authors regarding the inclusion of the study were resolved by a third author. Two authors extracted data independently, and disagreements were also resolved by a third author.

### Methodological Rigor of Included Studies and Certainty of Evidence

We assessed the methodological characteristics of the trials using the PEDro scale^[18]^. We used PEDro scores available at https://pedro.org.au/. Where PEDro scores were not available, two previously trained authors evaluated the clinical trials using the PEDro scale. The PEDro methodological rigor scale ranges between 1 and 10, with higher scores indicating higher quality studies. The studies are classified according to the scores as follows: < 4 are considered “poor”, 4 to 5 are considered “fair”, 6 to 8 are considered “good”, and 9 to 10 are considered “excellent”^[19]^. We assessed the certainty of evidence using the Classification of Recommendations, Assessment, Development and Evaluation (GRADE)^[20]^, through the software GRADEpro in the main outcomes^[21]^.

### Data Analysis

When at least two studies were sufficiently homogeneous in terms of participants, interventions, and outcome measures, we pooled their results in a meta-analysis. Meta-analyses were performed using an inverse variance method and random effects model in Review Manager version 5.3 (The Nordic Cochrane Center, Copenhagen, Denmark)^[22]^. Continuous variables were analyzed using the weighted mean differences (MD) and for studies that evaluated the same outcome with different instruments, we used the standardized mean differences (SMD) with 95% confidence interval (CI)^[23]^. Dichotomous variables were analyzed using risk ratios (RR) with 95% CI.

Trials were pooled according to similarity of intervention, populations, and the outcomes measured. Separate meta-analyses were conducted to examine the effects of IS in the following comparisons:

- IS *vs.* respiratory care.
- IS *vs.* NIV.
- IS *vs.* other therapies.

In case of trials that examined the effects of multiple interventions that were of interest for this review, to avoid double counting the participants, we included two reasonably independent comparisons. However, we split the “shared” group sample size (respiratory care) into two smaller sample sizes. For example, Stock et al. (1984)^[24]^ had three groups in its clinical trial: intervention group (with 12 participants), control group 1 (with 13 participants), and control group 2 (with 13 participants). In this situation, the analysis was performed twice; in the first analysis, the intervention group (with six participants [half the original sample size]) was analyzed *vs.* control group 1. In the second analysis, the intervention group (with six participants [half the original sample size]) was compared with control group 2.

Therefore, in the included clinical trials with three comparison groups (flow-IS group *vs.* volume-IS *vs.* respiratory care), and where data were analyzed twice in our study, we initially identified the name of the main author, and then the year of publication, followed by the letter “a” (Amin et al 2021a: flow-IS group *vs.* respiratory care) and in the second mention, we identified the name of the main author, and then the year of publication, followed by the letter “b” (Amin et al 2021b: volume-IS group *vs.* second standard care)^[25]^.

### Assessment of Heterogeneity

As planned, where appropriate data were available, we carried out subgroup analyses so as to investigate the influence of each comparison on the size of the treatment. Among the preplanned subgroup analyses, it was possible to perform subgroup analyses considering the type of device used (flow-oriented or volume-oriented) in the main comparisons (IS *vs.* respiratory care; IS *vs.* NIV; and IS *vs.* other therapies).

To estimate the heterogeneity across the studies in each meta-analysis, the I2 statistic was used. As suggested in the Cochrane Handbook for Systematic Reviews of Interventions, if heterogeneity was substantial (I2 ≥ 50%), a sensitivity analysis was considered^[26]^. Although we intended to perform separate analyses for studies with no blinding or deficiency in blinding of outcome assessors, with inappropriate randomization methods, with a large number (> 20%) of patients lost to follow-up, with imputation of standard deviation, or when adherence was not reported, we could not perform sensitivity analyses because we did not find enough studies with appropriate blinding, randomization, or follow-up.

## RESULTS

**Fig. 1:**
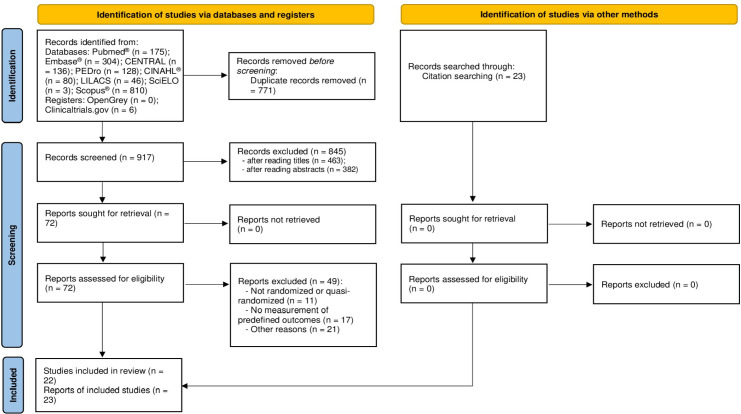
Flow diagram of systematic review. CENTRAL=Cochrane Central Register of Controlled Trials; CINAHL®=Cumulative Index of Nursing and Allied Health; LILACS=Latin American and Caribbean Health Sciences Literature; PEDro=Physiotherapy Evidence Database; SciELO=Scientific Electronic Library Online.

Twenty-three reports of 22 studies were included in this systematic review^[27-48]^. Twenty-two publications were reported in full; from one clinical trial, only the abstract was reported. One study with two publications was included in this systematic review. The reports of this study were named as Jenkins et al. (1989)^[30]^ and Jenkins et al. (1990)^[31]^, however, as planned, they were considered as part of only one study. The authors of the clinical trial published in abstract format were contacted in an attempt to request additional data^[39]^, however, we did not receive any answers. In this case, we used the data available in the abstract. Twenty-one studies were randomized controlled trials (RCTs) and one was a quasi-randomized trial. The flow chart of this systematic review is shown in Figure 1.

### Included Studies

Overall, we included 21 randomized trials and one quasi-randomized controlled trial in this systematic review. The studies involved 1,677 patients, with ages ranging from 38.3 to 65 years^[31,45]^, sample sizes ranging from 16 to 270 participants^[43,46]^, and study follow-up time ranging from two days to hospital discharge (Table 1)^[33,39,40,42]^. Regarding the characteristics of the surgery and intervention, 74% of patients underwent CABG, 48% of patients received treatment using volume-oriented IS, 39% of patients used flow-oriented IS, and three studies did not have enough information to determine whether the type of spirometer was flowor volume-oriented (Table 2)^[39,46,48]^. The hospital LOS ranged from 6.5 to 12.5 days, and the length of ICU stay ranged from 2.61 to 6.87 days (Table 3). PaO_2_ ranged on average from 59.4 mmHg to 99 mmHg^[24,28]^, and SO_2_ from 79 to 97.7%^[35,39]^.

**Table 1:** Characteristics of the studies included in the systematic review.

Study	Total sample size	Total average age (year)	Study follow-up time
Iverson et al., 1978^[27]^	145	-	-
Gale and Sanders, 1980^[28]^	109	-	Until 3^rd^ PO day, or longer if abnormal signs were present in the chest or on the chest X-ray
Dull and Dull, 1983^[29]^	49	57.9	Until 3^rd^ PO day
Stock et al., 1984^[24]^	38	57.3	Until 3^rd^ PO day
Jenkins et al., 1989^[30]^	110	55	Until 5^th^ PO day
Jenkins et al., 1990^[31]^	110	38.3	Until 5^th^ PO day
Oikkonen et al., 1991^[32]^	52	55	Until 7^th^ PO day
Crowe and Bradley, 1997^[33]^	185	64.4	Until hospital discharge
Savci et al., 2006^[34]^	60	56.2	Until 5^th^ PO day
Romanini et al., 2007^[35]^	40	56.75	Until 3^rd^ PO day
Renault et al., 2009^[36]^	36	56.8	Until 7^th^ PO day
Dias et al., 2011^[37]^	35	62.3	Until 5^th^ PO day
El-Kader, 2011^[38]^	36	48.6	Until 10^th^ PO day
Al-Mutairi et al., 2012a^[39]^	72	57	Until 2^nd^ PO day
Al-Mutairi et al., 2012b^[40]^	108	62	Until 2^nd^ PO day
Mueenudheen et al., 2012^[41]^	32	53.87	Until 3^rd^ PO day
Rizwan et al., 2012^[42]^	32	38.34	Until 2^nd^ PO day
Zangerolamo et al., 2013^[43]^	16	64.65	Until discharge from the intensive care unit
Yazdannik et al., 2016^[44]^	50	57.25	Until 3^rd^ PO day
Manapunsopee et al., 2019^[45]^	90	65	Until 4^th^ PO day
Alam et al., 2020^[46]^	270	46.9	Until 3^rd^ PO day
Amin et al., 2021^[47]^	72	62.56	Until 7^th^ PO day
Barkhordari-Sharifabad et al., 2021^[48]^	40	64.1	Until 4^th^ PO day

PO=postoperative

**Table 2:** Characteristics of the surgery and intervention.

Study	Type of surgery	ECC time (minutes)		Intervention with IS		Intervention with standard care			
		IS	Standard care	Technique	Frequency of use	Standard care 1		Standard care 2	
						Technique	Frequency of use	Technique	Frequency of use
Iverson et al., 1978^[27]^	CABG	-	-	Volume-oriented IS	Three to five times every 3 hours	IPPB	15-min. treatment every 3 hours with 15 to 20 cmH₂0	Blow bottles	Three to five times every 3 hours
Gale and Sanders, 1980^[28]^	CABG	-	-	Volume-oriented IS	10 deep breaths in a treatment time of 20 min.	IPPB	20 min. with inspiratory pressure of 20 cmH₂O	-	-
Dull and Dull, 1983^[29]^	CABG + VR	-		Volume-oriented IS	10 repetitions of maximal inhalation, four times a day	Early mobilization	Early mobilization twice a day	Early mobilization + respiratory care	10 repetitions of maximal inhalation four times a day
Stock et al., 1984^[24]^	CABG + VR + CABG and VR or aneurysmectomy	-		Volume-oriented IS	15 min. - every 2 hours during waking hours, from the second to the 72^nd^ hour after extubation	Coughing + deep breathing	15 min. - every 2 hours during waking hours, from the second to the 72^nd^ hour after extubation	CPAP	Pressure of 7.5 cmH₂O - two or three maximal inspirations every 3 to 5 min.
Jenkins et al., 1989^[30]^	CABG	-		Flow‑oriented IS	3 to 5 consecutive breaths, at least twice on days 1 and 2 and at least once daily on days 3 to 5	Respiratory care	3 to 5 consecutive deep breaths, at least twice on days 1 and 2 and at least once daily on days 3-5	Airway clearance exercises + physical therapy	3 to 5 consecutive deep breaths, at least twice on days 1 and 2 and at least once daily on days 3-5
Jenkins et al., 1990^[31]^	CABG	-		Flow‑oriented IS	Inspiration leaving the balls floating in the first and second chambers - 3 to 5 breaths	Respiratory care	Deep inspiration. Between 3 and 5 consecutive deep breaths	Verbal encouragement	Treatment consisted solely of encouraging (verbally) patients to huff and cough and early mobilization
Oikkonen et al., 1991^[32]^	CABG	96 ± 6	108 ± 6	Volume-oriented IS	Inhalation, exceeding 3 seconds and repeated at least 5 times per training	IPPB	Pressure of 10 to 15 cmH₂O - 5 to 10 min. in each session	-	-
Crowe and Bradley, 1997^[33]^	CABG	-	-	Volume-oriented IS	Provided once or twice per day, encouraged by other members of the health care team	Respiratory care	Lung expansion maneuvers and secretion-removal maneuvers. Provided once or twice per day	-	-
Savci et al., 2006^[34]^	CABG	-	-	Volume-oriented IS	Twice a day. From the 3^rd^ day, once a day for 15 min.	Respiratory care	1-2 breathing control breaths	-	-
Romanini et al., 2007^[35]^	CABG	57.50 ± 11.53	54 ± 9.26	Volume-oriented IS	10 min., an interval of 5 min. and 10 min. again.	IPPB	10 min., an interval of 5 min. and 10 min. again	-	-
Renault et al., 2009^[36]^	CABG	84.77 ± 32.29	80.94 ± 25.34	Flow‑oriented IS	2 times a day (ICU), and once a day (inpatient unit)	Respiratory care	3 sets of 10 breathing exercises + assisted cough and huffing + early mobilization	-	-
Dias et al., 2011^[37]^	CABG and VR	-	-	Volume-oriented IS	Twice a day for 5 days	Bronchial hygiene + mobilization	Twice a day for 5 days	Bronchial hygiene + mobilization + inspiratory exercise	Twice a day for 5 days
El-Kader, 2011^[38]^	CABG	-	-	Volume-oriented IS	Application of 5 min., five times a day	CPAP	Application of 15 min. and pressure = l0 cmH₂O every day	IPPB	Application of IPPB 15 min./day
Al-Mutairi et al., 2012a^[39]^	Any heart surgery	-	-	IS	Used IS 15 times per hour for 3 days	CPAP	4-6 cmH₂O	-	-
Al-Mutairi et al., 2012b^[40]^	Any heart surgery	-	-	Volume-oriented IS	15 times per hour for 3 days	CPAP (2 hours)	4-6 cmH₂O for half hour every 2 hours for 3 days	CPAP (4 hours)	4-6 cmH₂O for half hour every 4 hours for 3 days
Mueenudheen et al., 2012^[41]^	CABG	-	-	Flow‑oriented IS	3 sets of 10 breaths with a pause of 1 min. between each set	Respiratory care	3 sets of 10 consecutive breaths with a pause of 1 min. between each set	-	-
Rizwan et al., 2012^[42]^	Mitral valve replacement surgery	-	-	Flow‑oriented IS	3 sets of 10 deep breaths with 30-60 seconds to rest	Respiratory care	3 sets of 10 deep breaths with 30-60 seconds to rest	-	-
Zangerolamo et al., 2013^[43]^	CABG	64.3 ± 11.1	57.5 ± 10	Flow‑oriented IS	3 sets of 10 repetitions	Respiratory care	For each exercise, three sets of 10 repetitions. 3 sessions per day	-	-
Yazdannik et al., 2016^[44]^	CABG	-	-	Flow‑oriented IS	10 times breathing with IS every 2 hours in the daytime for three days	Respiratory care	Only usual exercise	-	-
Manapunsopee et al., 2019^[45]^	CABG	109.0 ± 55.0	129.0 ± 57.0	Flow‑oriented IS + respiratory care	10 times per hour - slow maximal inhalations	Respiratory care	Breathing exercise 10 times per hour	-	-
Alam et al., 2020^[46]^	CABG	-	-	IS	Breathing exercise with IS	Standard physiotherapy + Acapella	Breathing exercise + Acapella	-	-
Amin et al., 2021^[47]^	CABG	-	-	Flow‑oriented IS	3 sets of 5 repeated deep breaths - four times a day	Volume-oriented IS	3 sets of 5 repeated deep breaths - four times in a day	Respiratory care	3 sets of 5 deep breaths - 4 times in a day
Barkhordari-Sharifabad et al., 2021^[48]^	CABG	-	-	IS	10 deep breaths, every 2 hours during awakening	Respiratory care	10 times with 2-hour interval when the patient woke up	-	-

CABG=coronary artery bypass grafting; CPAP=continuous positive airway pressure; ECC=extracorporeal circulation; ICU=intensive care unit; IPPB=intermittent positive pressure breathing; IS=incentive spirometry; VR=valve replacement

**Table 3:** Summary of findings for clinical outcomes.

Study	Outcomes observed									
	PPC (n,%)		Adverse events (n,%)		Mortality (n,%)		Length of hospital stay (days) (mean ± SD)		Length of intensive care unit stay (days) (mean ± SD)	
	ISG	CG	ISG	CG	ISG	CG	ISG	CG	ISG	CG
Iverson et al., 1978a^[27]^	35 (60.3)^*^	23 (54.7)^*^ and 1 (2.3)^**^	0	1 (2.4)	0	1 (2.4)	-	-	-	-
Iverson et al., 1978b^[27]^	35 (60.3)^*^	18 (40)^*^	0	0	0	0	-	-	-	-
Gale and Sanders, 1980^[28]^	51 (98)^*^	57 (100)^*^	-	-	-	-	-	-	-	-
Dull and Dull, 1983^[29]^	-	-	-	-	-	-	-	-	-	-
Stock et al., 1984a^[24]^	11 (92)^*^	12 (92)^*^	0 (0)	1 (8)	-	-	-	-	-	-
Stock et al., 1984b^[24]^	11 (92)^*^	9 (67)^*^	0 (0)	2 (15)	-	-	-	-	-	-
Jenkins et al., 1989a^[30]^	28 (74)^*^ and 2 (5.2)^**^	26 (74)^*^ and 4 (11.4)^**^	-	-	-	-	-	-	-	-
Jenkins et al., 1989b^[30]^	28 (74)^*^ and 2 (5.2)^**^	28 (75)^*^ and 5 (13.5)^**^	-	-	-	-	-	-	-	-
Jenkins et al., 1990a^[31]^	28 (74)^*^ and 2 (5.2)^**^	26 (74)^*^ and 4 (11.4)^**^	-	-	-	-	-	-	-	-
Jenkins et al., 1990b^[31]^	28 (74)^*^ and 2 (5.2)^**^	28 (75)^*^ and 5 (13.5)^**^	-	-	-	-	-	-	-	-
Oikkonen et al., 1991^[32]^	21 (80.7)^*^	16 (61.5)^*^	11 (24.3)	13 (50)	-	-	-	-	-	-
Crowe and Bradley, 1997^[33]^	9 (10)^*^ and 8 (8.9)^**^	10 (10.5)^*^ and 10 (10.5)^**^	-	-	-	-	9 ± 3.1	9.7 ± 4.9	-	-
Savci et al., 2006^[34]^	9 (30)^*^	10 (33.3)^*^	-	-	-	-	-	-	-	-
Romanini et al., 2007^[35]^	-	-	-	-	-	-	-	-	-	-
Renault et al., 2009^[36]^	-	-	-	-	-	-	-	-	2.61 ± 0.69	3.22 ± 1.06
Dias et al., 2011a^[37]^	-	-	0 (0)	0 (0)	-	-	-	-	-	-
Dias et al., 2011b^[37]^	-	-	0 (0)	0 (0)	-	-	-	-	-	-
El-Kader, 2011^[38]^	-	-	-	-	-	-	-	-	-	-
Al-Mutairi et al., 2012a^[39]^	-	-	-	-	-	-	-	-	-	-
Al-Mutairi et al., 2012b1^[40]^	-	-	-	-	0 (0)	1 (2.8)	9.5 ± NR	8.7 ± NR	-	-
Al-Mutairi et al., 2012b2^[40]^	-	-	-	-	0 (0)	2 (5.6)	9.5 ± NR	9 ± NR	-	-
Mueenudheen et al., 2012^[41]^	-	-	-	-	-	-	-	-	-	-
Rizwan et al., 2012^[42]^	-	-	-	-	-	-	-	-	-	-
Zangerolamo et al., 2013^[43]^	-	-	-	-	1 (12.5)	2 (25)	6.5 ± 1.69	8.25 ± 2.6	5 ± 1.6	6.87 ± 2.74
Yazdannik et al., 2016^[44]^	-	-	-	-	-	-	-	-	-	-
Manapunsopee et al., 2019^[45]^	3 (6.4)^*^ and 1 (2.1)^**^	5 (11.6)^*^	17 (36.2)	10 (23.2)	-	-	6.75 ± 7.77	12.5 ± 18.82	-	-
Alam et al., 2020^[46]^	-	-	-	-	-	-	-	-	-	-
Amin et al., 2021^[47]^	-	-	-	-	-	-	-	-	-	-
Barkhordari-Sharifabad et al., 2021^[48]^	-	-	-	-	-	-	-	-	-	-

CG=control group; ISG=incentive spirometry group; NR=not registered; PPC=postoperative pulmonary complications; SD=standard deviation

* Atelectasis

** Pneumonia

Iverson et al. 1978a=comparison of intervention group *vs.* first control group; Iverson et al. 1978b=comparison of intervention group *vs.* second control group; Stock et al. 1984a=comparison of intervention group *vs.* first control group; Stock et al. 1984b=comparison of intervention group *vs.* second control group; Jenkins et al. 1989a=comparison of intervention group *vs.* first control group; Jenkins et al. 1989b=comparison of intervention group *vs.* second control group; Jenkins et al. 1990a=comparison of intervention group *vs.* first control group; Jenkins et al. 1990b=comparison of intervention group *vs.* second control group; Dias et al. 2011a=comparison of intervention group *vs.* first control group; Dias et al. 2011b=comparison of intervention group *vs.* second control group; Al-Mutairi et al. 2012b1=comparison of intervention group *vs*. first control group; Al-Mutairi et al. 2012b2=comparison of intervention group *vs.* second control group

Considering the primary outcomes analysis, among the included studies, nine clinical trials reported PPC rate^[24,27,28,30-34,45]^, five reported adverse events rate^[24,27,32,37,45]^, and three reported mortality rate (Table 3)^[26,40,43]^.

With respect to the secondary outcomes analysis, four trials reported LOS^[33,40,43,45]^, two reported ICU LOS^[36,43]^, eight reported parameters of lung function^[24,29,30,33,34,36,41,47]^, ten reported PaO_2_, nine reported SO_2_, and one reported reintubation rate. No trials evaluated the use of antibiotics (which was an outcome of interest for this review^[16]^).

For these continuous outcomes, results were reported differently across studies, and we performed transformations where it was adequate. In two clinical trials, PaO_2_ was converted from kilopascals to millimeters of mercury and in one clinical trial the standard deviation was estimated using the Revman calculator^[28,31,32]^. For some studies the standard deviation was also estimated using the Revman calculator^[24,37,39,47]^. In one clinical trial, LOS was registered as median, with minimum and maximum, and this was converted to mean and standard deviation for our analysis^[45,49]^ (Table 4). For some studies, transformations were not possible. For instance, one clinical trial recorded forced expiratory flow without standard deviation^[29]^, and insufficient information to estimate the standard deviation. Therefore, we did not pool the results in the meta-analysis. When results were presented using different measures, such as those from studies reporting lung function, which reported values both as a percentage of predicted values and as absolute values in liters, then results were pooled using the SMD.

**Table 4:** Summary of findings for clinical outcomes.

	Lung function								Oxygenation					
Study	FEV1%		FVC%		PEF (L/min)		VC (%)		FEF (%)		PaO2 (mmHg)		SO2 (%)	
	ISG	CG	ISG	CG	ISG	CG	ISG	CG	ISG	CG	ISG	CG	ISG	CG
Iverson et al., 1978a^[27]^	-	-	-	-	-	-	-	-	-	-	-	-	-	-
Iverson et al., 1978b^[27]^	-	-	-	-	-	-	-	-	-	-	-	-	-	-
Gale and Sanders, 1980^[28]^	-	-	-	-	-	-	1.8 ± 1.4 L	1.4 ± 1.5 L	-	-	60.6 ± 13.7	59.4 ± 12.07	-	-
Dull and Dull, 1983a^[29]^	37 ± NR	40 ± NR	35 ± NR	35 ± NR	-	-	-	-	55 ± NR	70 ± NR	-	-	-	-
Dull and Dull, 1983b^[29]^	37 ± NR	40 ± NR	35 ± NR	41 ± NR	-	-	-	-	55 ± NR	53 ± NR	-	-	-	-
Stock et al., 1984a^[24]^	0.76 ± 0.173 L	0.63 ± 0.288 L	0.96 ± 0.243 L	0.76 ± 0.324 L	-	-	-	-	-	-	93 ± 17	99 ± 21	-	-
Stock et al., 1984b^[24]^	0.76 ± 0.173 L	0.59 ± 0.252 L	0.96 ± 0.243 L	0.73 ± 0.283 L	-	-	-	-	-	-	93 ± 17	94 ± 17	-	-
Jenkins et al., 1989a^[30]^	1.8 ± 0.5 L	1.7 ± 0.4 L	2.4 ± 0.5 L	2.2 ± 0.5 L	2.55 ± 0.6	2.50 ± 0.6	-	-	-	-	60 ± 6.7	67.5 ± 7.5	-	-
Jenkins et al., 1989b^[30]^	1.8 ± 0.5 L	1.8 ± 0.5 L	2.4 ± 0.5 L	2.3 ± 0.6 L	2.55 ± 0.6	2.53 ± 0.67	-	-	-	-	60 ± 6.7	66.7 ± 7.5	-	-
Jenkins et al., 1990a^[31]^	-	-	-	-	-	-	2.6 ± 0.1 L	2.5 ± 0.1 L	-	-	60 ± 7.5	60 ± 7.5	-	-
Jenkins et al., 1990b^[31]^	-	-	-	-	-	-	2.6 ± 0.1 L	2.7 ± 0.2 L	-	-	60 ± 7.5	60 ± 7.5	-	-
Oikkonen et al., 1991^[32]^	-	-	-	-	-	-	-	-	-	-	75 ± 7.5	82.5 ± 7.5	-	-
Crowe and Bradley, 1997^[33]^	81 ± 4	83 ± 4	82 ± 6	85 ± 4	-	-	-	-	-	-	-	-	90 ± NR	79 ± NR
Savci et al., 2006^[34]^	57.26 ± 14.6	64.98 ± 12.95	57.6 ± 14.17	63.17 ± 11.65	4.54 ± 1.44	5.90 ± 1.96	53.18 ± 13.6	57.76 ± 9.47	-	-	76.08 ± 13.69	79.69 ± 18.26	95.74 ± 2.13	94.59 ± 4.33
Romanini et al., 2007^[35]^	-	-	-	-	-	-	-	-	-	-	-	-	91.15 ± 83.2	94.7 ± 86.4
Renault et al., 2009^[36]^	1.12 ± NR L	1.37 ± NR L	1.37 ± NR L	1.27 ± NR L	-	-	-	-	-	-	-	-	-	-
Dias et al., 2011a^[37]^	-	-	46.7 ± 52.08	51.3 ± 37.09	-	-	-	-	-	-	-	-	97.2 ± NR	97 ± NR
Dias et al., 2011b^[37]^	-	-	46.7 ± 52.08	54.3 ± 49.64	-	-	-	-	-	-	-	-	97.2 ± NR	97.7 ± NR
El-Kader, 2011a^[38]^	-	-	-	-	-	-	-	-	-	-	82.91 ± 2.3	71.66 ± 4	-	-
El-Kader, 2011b^[38]^	-	-	-	-	-	-	-	-	-	-	82.91 ± 2.3	74.5 ± 4.8	-	-
Al-Mutairi et al., 2012a^[39]^	-	-	-	-	-	-	1.59 ± 3.9 L	1.88 ± 4.6 L	-	-	-	-	96.53 ± 181.5	96.83 ± 182.1
Al-Mutairi et al., 2012b1^[40]^	-	-	-	-	-	-	-	-	-	-	-	-	95.6 ± 0.4	97.17 ± 0.43
Al-Mutairi et al., 2012b2^[40]^	-	-	-	-	-	-	-	-	-	-	-	-	95.6 ± 0.4	96 ± 0.6
Mueenudheen et al., 2012^[41]^	52 ± 10	54 ± 9	46 ± 9	45 ± 8	-	-	-	-	-	-	79.77 ± 9.22	82.85 ± 10.53	-	-
Rizwan et al., 2012^[42]^	-	-	-	-	-	-	-	-	-	-	76.41 ± 15.85	75.35 ± 11.55	94.72 ± 2.9	94.7 ± 1.95
Zangerolamo et al., 2013^[43]^	-	-	-	-	-	-	1.68 ± 0.48 L	1.4 ± 0.4 L	-	-	-	-	-	-
Yazdannik et al., 2016^[44]^	-	-	-	-	-	-	-	-	-	-	72.7 ± 7.1	82.3 ± 4.8	96.8 ± 1.4	90.5 ± 2.1
Manapunsopee et al., 2019^[45]^	-	-	-	-	-	-	-	-	-	-	-	-	-	-
Alam et al., 2020^[46]^	-	-	-	-	-	-	-	-	-	-	-	-	-	-
Amin et al., 2021a^[47]^	0.96 ± 1.2 L	0.86 ± 1.1 L	1.10 ± 1.4 L	0.90 ± 1.1 L	1.44 ± 1.8 L	1.08 ± 1.4 L	-	-	-	-	-	-	-	-
Amin et al., 2021b^[47]^	1.26 ± 15.2 L	0.86 ± 1.1 L	1.53 ± 1.9 L	0.90 ± 1.1 L	2.22 ± 44.7 L	1.08 ± 1.4 L	-	-	-	-	-	-	-	-
Barkhordari-Sharifabad et al., 2021^[48]^	-	-	-	-	-	-	-	-	-	-	-	-	96.5 ± 1.50	95.2 ± 2.38

CG=control group; FEF=forced expiratory flow; FEV₁=forced expiratory volume in one second; FVC=forced vital capacity; ISG=incentive spirometry group; NR=not registered; PaO_2_=partial pressure of oxygen; PEF=peak of expiratory flow; SO_2_=oxygen saturation; VC=vital capacity

Iverson et al. 1978a=comparison of intervention group *vs*. first control group; Iverson et al. 1978b=comparison of intervention group *vs*. second control group; Dull and Dull 1983a=comparison of intervention group *vs*. first control group; Dull and Dull 1983b=comparison of intervention group *vs*. second control group; Stock et al. 1984a=comparison of intervention group *vs*. first control group; Stock et al. 1984b=comparison of intervention group *vs*. second control group; Jenkins et al. 1989a=comparison of intervention group *vs*. first control group; Jenkins et al. 1989b=comparison of intervention group vs. second control group; Jenkins et al. 1990a=comparison of intervention group *vs*. first control group; Jenkins et al. 1990b=comparison of intervention group *vs*. second control group; Dias et al. 2011a=comparison of intervention group *vs*. first control group; Dias et al. 2011b=comparison of intervention group *vs*. second control group; El-Kader 2011a=comparison of intervention group *vs*. first control group; El-Kader 2011b=comparison of intervention group *vs*. second control group; Al-Mutairi et al. 2012b1=comparison of intervention group *vs*. first control group; Al-Mutairi et al. 2012b2=comparison of intervention group *vs*. second control group; Amin et al. 2021a=comparison of intervention group *vs*. first control group; Amin et al. 2021b=comparison of intervention group *vs*. second control group

### Assessment of Methodological Rigor

Among the included studies, in general, the PEDro score ranged from 2 to 7 points, with a mean and standard deviation of 4.5±1.1. For seven trials, the scores were not available on the PEDro platform, therefore, the scores were independently graded by two authors^[27,39,42-44,47,48]^. After the evaluation of the two authors, three inconsistencies were observed, one on item 11 and two on item 8^[27,42,44]^. In this situation, a third author was consulted to arbitrate. Considering the PEDro scale, the following percentages of studies did not meet the criteria: on item 1, 21.7%; on item 2, 8.7%; on item 3, 95.7%; on item 4, 13%; on items 5 and 6, 100%; on item 7, 78.2%; on item 8, 52.1%; on item 9, 95.7%; and on item 11, 8.7%. On item 10, all studies met the criteria. In the classification of the PEDro scale, 16 (69.6%) studies were judged as having “fair”^[24,27,28,30,31,35,37-44,47,48]^, four (17.4%) as “good”^[32-34,45]^, three (13%) as “poor”^[29,36,46]^, and zero (0%) as “excellent” quality (Table 5). Considering the low methodological rigor of the studies included in this review, we were not able to perform sensitivity analysis including only high-quality studies.

**Table 5:** Quality assessment of the clinical trials using the Physiotherapy Evidence Database (or PEDro) scale.

Study	Total score	Item 1	Item 2	Item 3	Item 4	Item 5	Item 6	Item 7	Item 8	Item 9	Item 10	Item 11
Iverson et al., 1978^[27]^	4^*^	N	N	N	Y	N	N	N	Y	N	Y	Y
Gale and Sanders, 1980^[28]^	4	Y	Y	N	N	N	N	N	Y	N	Y	Y
Dull and Dull, 1983^[29]^	3	N	Y	N	Y	N	N	N	N	N	Y	N
Stock et al., 1984^[24]^	5	N	Y	N	Y	N	N	N	Y	N	Y	Y
Jenkins et al., 1989^[30]^	5	Y	Y	N	Y	N	N	Y	N	N	Y	Y
Jenkins et al., 1990^[31]^	4	Y	Y	N	Y	N	N	N	N	N	Y	Y
Oikkonen et al., 1991^[32]^	6	Y	Y	N	Y	N	N	Y	Y	N	Y	Y
Crowe and Bradley, 1997^[33]^	6	Y	Y	N	Y	N	N	Y	Y	N	Y	Y
Savci et al., 2006^[34]^	6	Y	Y	N	Y	N	N	Y	Y	N	Y	Y
Romanini et al., 2007^[35]^	4	N	Y	N	Y	N	N	N	N	N	Y	Y
Renault et al., 2009^[36]^	2	Y	N	N	Y	N	N	N	N	N	Y	N
Dias et al., 2011^[37]^	4	Y	Y	N	Y	N	N	N	N	N	Y	Y
El-Kader, 2011^[38]^	5	N	Y	N	Y	N	N	N	Y	N	Y	Y
Al-Mutairi et al., 2012a^[39]^	5^*^	Y	Y	N	Y	N	N	N	Y	N	Y	Y
Al-Mutairi et al., 2012b^[40]^	4	Y	Y	N	N	N	N	N	Y	N	Y	Y
Mueenudheen et al., 2012^[41]^	4	Y	Y	N	Y	N	N	N	N	N	Y	Y
Rizwan et al., 2012^[42]^	4^*^	Y	Y	N	Y	N	N	N	N	N	Y	Y
Zangerolamo et al., 2013^[43]^	4^*^	Y	Y	N	Y	N	N	N	N	N	Y	Y
Yazdannik et al., 2016^[44]^	4^*^	Y	Y	N	Y	N	N	N	N	N	Y	Y
Manapunsopee et al., 2019^[45]^	7	Y	Y	Y	Y	N	N	Y	N	Y	Y	Y
Alam et al., 2020^[46]^	3	Y	Y	N	N	N	N	N	N	N	Y	Y
Amin et al., 2021^[47]^	5^*^	Y	Y	N	Y	N	N	N	Y	N	Y	Y
Barkhordari-Sharifabad et al., 2021^[48]^	5^*^	Y	Y	N	Y	N	N	N	Y	N	Y	Y

N=no; Y=yes

* =Score assessed by authors

Item 1=eligibility criteria were specified (item 1 is not included in the total score calculation); item 2=subjects were randomly allocated to groups (in a crossover study, subjects were randomly allocated an order in which treatments were received); item 3=allocation was concealed; item 4=the groups were similar at baseline regarding the most important prognostic indicators; item 5=there was blinding of all subjects; item 6=there was blinding of all therapists who administered the therapy; item 7=there was blinding of all assessors who measured at least one key outcome; item 8=measures of at least one key outcome were obtained from more than 85% of the subjects initially allocated to groups; item 9=all subjects for whom outcome measures were available received the treatment or control condition as allocated or, where this was not the case, data for at least one key outcome was analyzed by “intention to treat”; item 10=the results of between-group statistical comparisons are reported for at least one key outcome; item 11=the study provides both point measures and measures of variability for at least one key outcome

### Comparisons of Interventions

We rated the certainty of the evidence for each outcome in all comparisons using the GRADE approach^[21]^. The details of each evaluation can be found in Supplement 2.

### Incentive Spirometry *vs.* Respiratory Care

#### Primary Outcomes

**Fig. 2:**
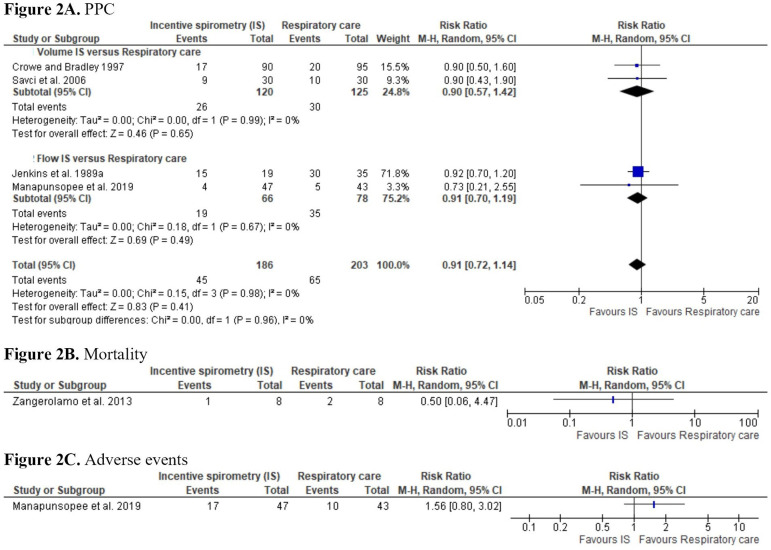
Meta-analyses of the results for the primary outcomes of incentive spirometry vs. respiratory care. CI=confidence interval; PPC=postoperative pulmonary complications

There may be a small difference or no difference on PPC rate between IS and respiratory care (RR 0.91; 95% CI 0.72 to 1.14) (low certainty of evidence) (Supplement 3 - Figure 2A). The evidence is of very low certainty for the other primary outcomes. Only one trial evaluated the mortality rate^[43]^. This trial also used flow IS and compared its effects to the effects of respiratory care (Supplement 3 - Figure 2B). In the same way, only one trial evaluated the adverse events^[45]^. This trial used flow IS and compared its effects to the effects of respiratory care (Supplement 3 - Figure 2C).

#### Secondary Outcomes

**Fig. 3:**
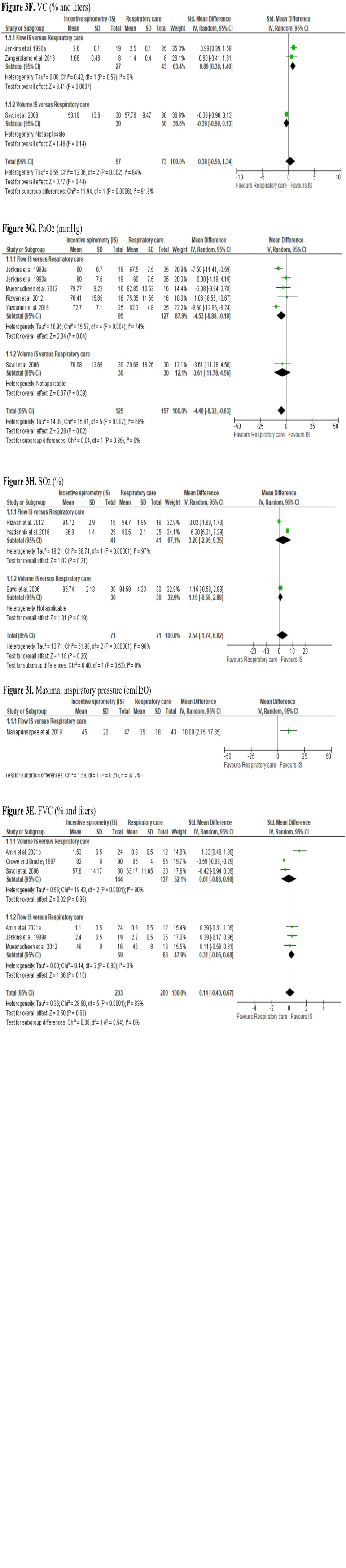
Meta-analyses of the results for the secondary outcomes of incentive spirometry vs. respiratory care. CI=confidence interval; FEV1=forced expiratory volume in one second; FVC=forced vital capacity; ICU=intensive care unit; LOS=length of stay; PaO_2_=partial pressure of oxygen; PEF=peak of expiratory flow; SD=standard deviation; SO_2_=oxygen saturation; VC=vital capacity

We found low certainty of evidence that there may be a small or no difference on FEV1 between IS and respiratory care (SMD -0.16; 95% CI -0.48 to 0.16) (Supplement 3 - Figure 3D). The evidence is of very low certainty for all the other secondary outcomes of this comparison. For these outcomes, no differences in LOS (MD -1.38; 95% CI -2.96 to 0.21), length of ICU stay (MD -0.78; 95% CI -1.61 to 0.06), PEF (MD -0.60; 95% CI -1.97 to 0.78), FVC (SMD 0.14; 95% CI -0.40 to 0.67), VC (SMD 0.38; 95% CI -0.59 to 1.34), and SO_2_ (MD 2.54; 95% CI -1.74 to 6.82) were observed, comparing IS and respiratory care (Supplement 3 - Figures 3A, 3B, 3C, 3E, 3F, and 3H). However, in the subgroup analysis of VC, flow IS was superior compared to respiratory care. The Amin et al. (2021)^[47]^ study was not included in the PEF meta-analysis as it did not have sufficient extractable data. The Barkhordari-Sharifabad et al. (2021)^[48]^ study was not included in the SO_2_ meta-analysis as it was unclear whether it used flow-oriented IS or volume-oriented IS.

The meta-analysis showed that IS leads to lower recovery of PaO_2_ than respiratory care (MD -4.48; 95% CI -8.32 to -0.63) (very low certainty of evidence). In the subgroup analyses, flow-oriented IS was inferior to recovery PaO_2_ compared to respiratory care (Supplement 3 - Figure 3G). Two trials evaluated MIP^[36,45]^, however, only one had sufficient extractable data (Supplement 3 - Figure 3I)^[45]^.

### Incentive Spirometry *vs.* Other Therapies

#### Primary Outcomes

**Fig. 4:**
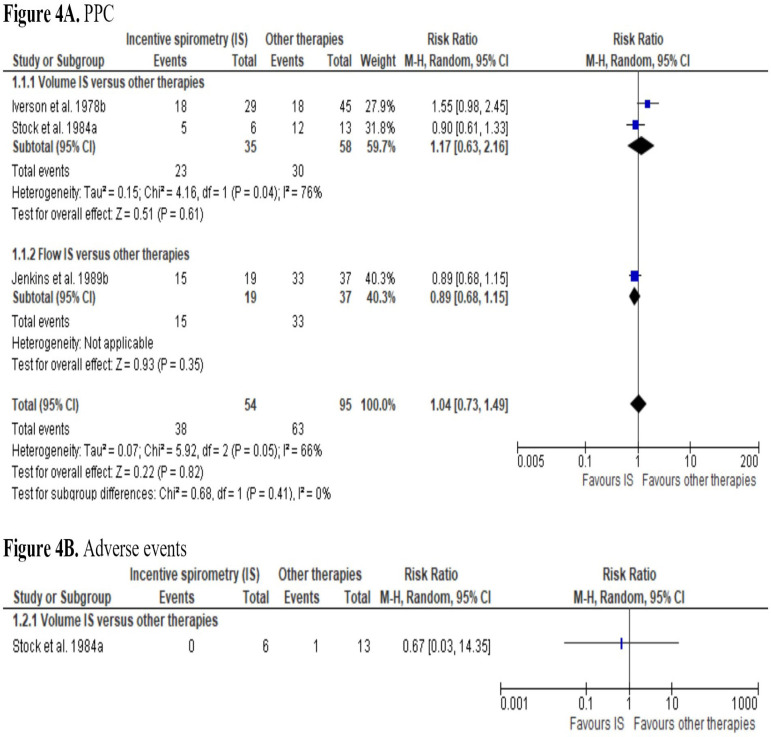
Meta-analyses of the results for the primary outcomes of incentive spirometry vs. other therapies. CI=confidence interval; PPC=postoperative pulmonary complications

The evidence for the primary outcomes of IS *vs.* other therapies is of very low certainty. We found no differences on PPC between IS and other therapies (RR 1.04; 95% CI 0.73 to 1.49) (Supplement 3 - Figure 4A). Only one study evaluated adverse events (Supplement 3 - Figure 4B).

#### Secondary Outcomes

**Fig. 5:**
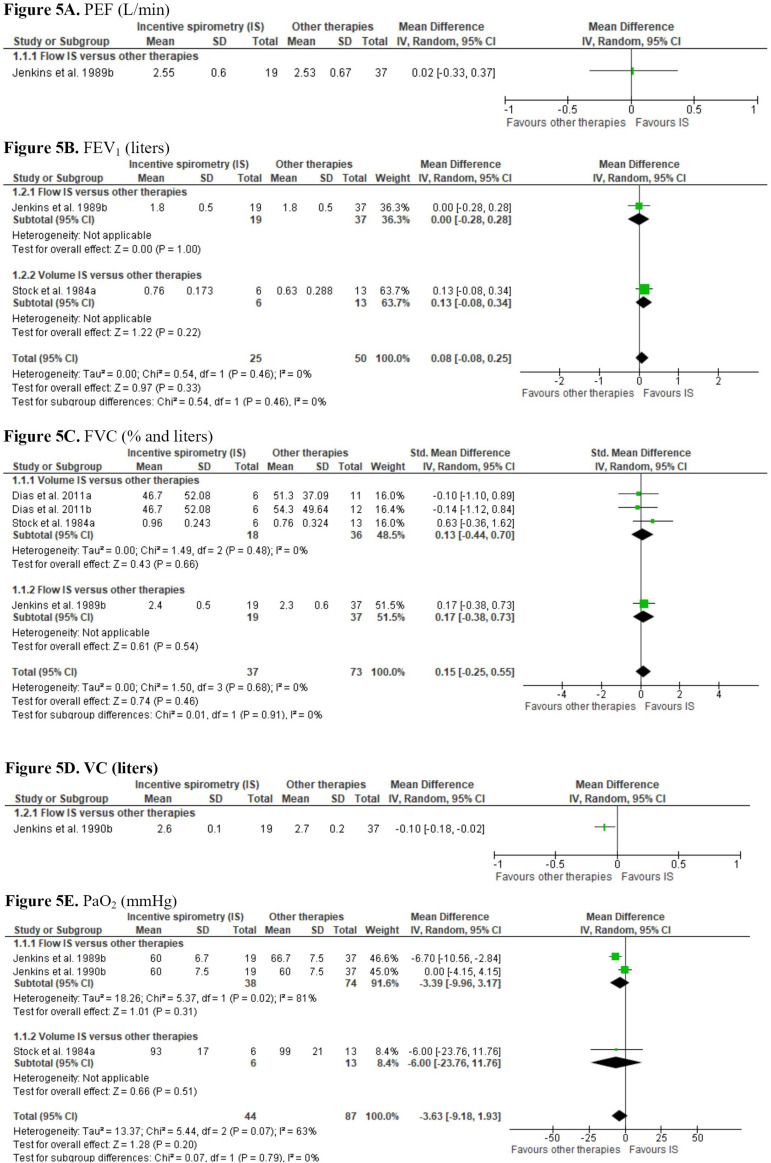
Meta-analyses of the results for the secondary outcomes of incentive spirometry vs. other therapies. CI=confidence interval; FEV1=forced expiratory volume in one second; FVC=forced vital capacity; PaO2=partial pressure of oxygen; PEF=peak of expiratory flow; SD=standard deviation; VC=vital capacity

The evidence for the secondary outcomes is also of very low certainty. No difference was observed between IS and other therapies regarding FEV1 (MD 0.08; 95% CI -0.08 to 0.25), FVC (SMD 0.15; 95% CI -0.25 to 0.55), and PaO2 (MD -3.63; 95% CI -9.18 to 1.93) (very low certainty of evidence) (Supplement 3 - Figures 5B, 5C, and 5E). Only one study evaluated PEF^[30]^, and another study evaluated VC (Supplement 3 - Figure 5A, 5D)^[31]^.

### Incentive Spirometry *vs.* NIV

#### Primary Outcomes

**Fig. 6:**
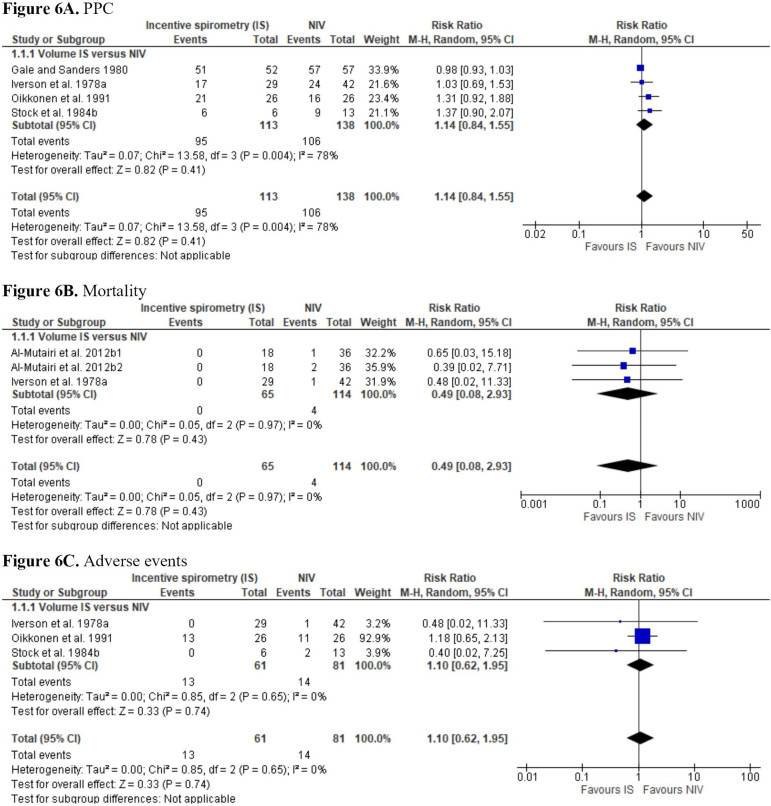
Meta-analyses of the results for the primary outcomes of incentive spirometry vs. noninvasive ventilation (NIV). CI=confidence interval; PPC=postoperative pulmonary complications

Four trials compared the effects of IS *vs.* NIV on PPC, and three trials on mortality and adverse events. The evidence for the primary outcomes of IS *vs.* NIV is of very low certainty. All trials used volume-oriented IS. No differences were found between volume-oriented IS and NIV on PPC (RR 1.14; 95% CI 0.84 to 1.55), mortality (RR 0.49; 95% CI 0.08 to 2.93), and adverse events (RR 1.10; 95% CI 0.62 to 1.95) (Supplement 3 - Figures 6A, 6B, and 6C).

#### Secondary Outcomes

**Fig. 7:**
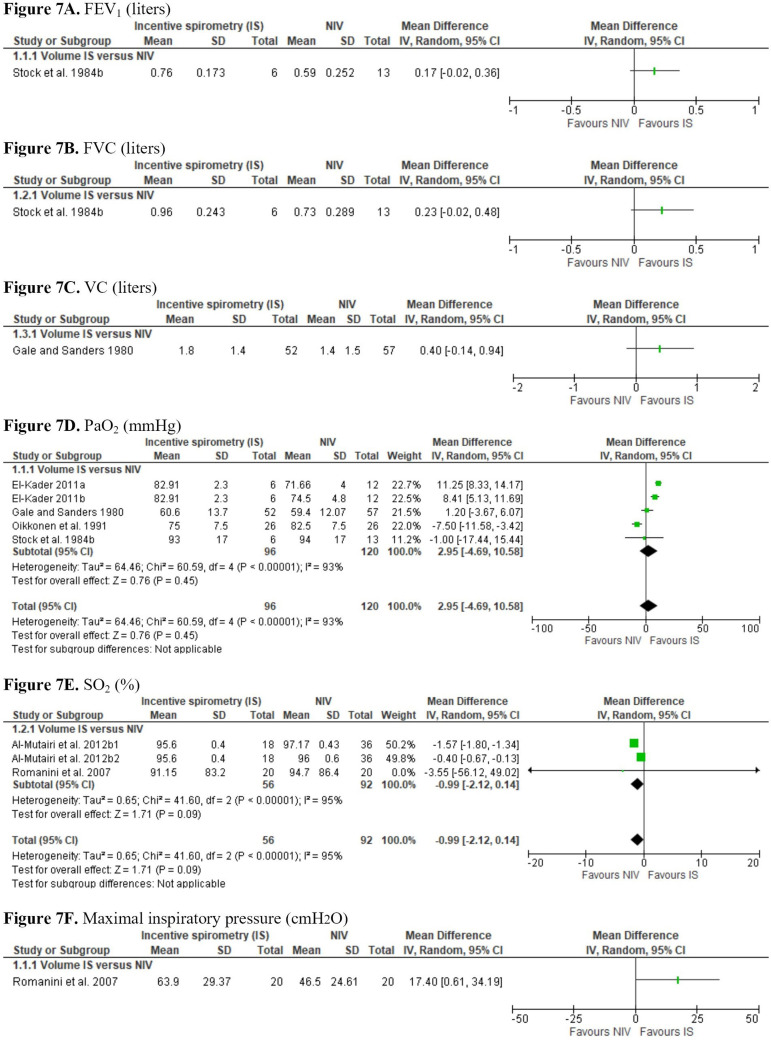
Meta-analyses of the results for the secondary outcomes of incentive spirometry vs. noninvasive ventilation (NIV). CI=confidence interval; FEV₁=forced expiratory volume in one second; FVC=forced vital capacity; PaO_2_=partial pressure of oxygen; SD=standard deviation; SO_2_=oxygen saturation; VC=vital capacity

The evidence for secondary outcomes is also of very low certainty. No differences were found between IS and NIV on PaO_2_ (MD 2.95; 95% CI -4.69 to 10.58) or on SO_2_ (MD -0.99; 95% CI -2.12 to 0.14) (Supplement 3 - Figures 7D, 7E). Only one trial compared the effects of IS and NIV on FEV1, FVC, VC, and MIP. All trials used volume-oriented IS (Supplement 3 - Figures 7A, 7B, 7C, and 7F). A single study recorded the reintubation rate, with zero reintubation in the IS group and one reintubation in the standard care group^[32]^.

## DISCUSSION

To the best of our knowledge, this is the first systematic review with meta-analysis to investigate the effects of IS exclusively in patients undergoing cardiac surgery, performing sub-analysis to pool the studies according to the type of IS used as respiratory care. The results showed that the use of IS was not superior to respiratory care, other therapies, and NIV on the outcomes evaluated. On the other hand, IS was inferior to respiratory care for recovery PaO_2_. In the subgroup analysis, flow-oriented IS was inferior to respiratory care on recovery PaO_2_. However, flow-oriented IS was superior to respiratory care on VC. Overall, the methodological rigor of the clinical trials included in this review was “fair” and the certainty of evidence ranged from “very low” to “low”.

In general, although our meta-analysis showed that IS is not different from respiratory care, other therapies, or NIV, except for PaO_2_ (in IS *vs.* respiratory care) for which we cannot make any positive or negative statements about effectiveness after cardiac surgery, the majority of the included studies present severe methodological problems and inadequate sample size. In addition, over the years studies have investigated the effects of IS on PPC, adverse events, and mortality after surgical procedures on the thorax, showing different results, some in agreement with and others contrary to our findings^[11,50,51]^.

Our results are in line with a previous Cochrane systematic review that included seven RCTs with a total of 592 patients to assess the effects of IS for preventing pulmonary complications after CABG^[51]^. This review found no evidence of a benefit from IS in reducing pulmonary complications and in decreasing the negative effects on pulmonary function in patients undergoing CABG. Of note, besides including only patients that had undergone CABG, this review is outdated and did not perform the certainty of evidence evaluation. The inclusion of a broader and updated body of knowledge and GRADE assessments in our review is of particular importance, as it facilitates decision making of physiotherapists working in the frontline.

A clinical trial investigated the effects of IS after cardiac surgery in 90 patients; 47 patients were treated with flow-oriented IS + deep breathing exercise, and 43 patients received only deep breathing exercise (control group)^[45]^. Patients who received IS + deep breathing exercise had no reduction in atelectasis, pneumonia, pneumothorax, and pleural effusion. However, the control group had fewer adverse events (dyspnea) (*P*-value = 0.03)^[45]^. On the other hand, one thing is certain, although, to date, the clinical efficacy on PPC is not proven, IS is widely used and investigated^[52]^.

A preliminary trial^[53]^ that investigated the effectiveness of IS (flow-oriented device) on respiratory motion in healthy subjects suggested that two weeks of respiratory training using IS is useful for improving respiratory motion and pulmonary function. A clinical trial^[54]^ with 260 surgical patients (non-cardiac patients) showed that IS (flow-oriented and volume-oriented) and diaphragmatic breathing exercise better preserve pulmonary function and diaphragm excursion. If these findings are also demonstrated in patients after cardiac surgery using IS, this method will represent an easily accessible and low-cost device to be used in the treatment of these patients.

A broad range of different types of IS devices and treatment protocols were used in the studies included in this review. However, we were unable to determine which of them is more effective. Although we planned to perform other subgroup analyses, we were also unable to identify whether the type of surgery, the severity of the disease, or details of the intervention, such as frequency, duration, and time the intervention started could influence the effect of intervention. Due to the heterogeneity of the RCTs regarding the combinations of interventions and comparisons, different comparisons had to be made, and we were only able to perform a few comprehensive meta-analyses. Therefore, the precision of effect estimates was jeopardized.

Furthermore, due to several methodological limitations in the included studies and conflicting results, further well-designed trials, with long-term follow-up, and which report the rate of core outcome results, such as PPC, adverse events, mortality, lung function, and LOS, are needed, as well as in the ICU. New RCTs should be standardized to provide more homogeneous and reliable data to properly compare the results. For example, studies should evaluate the same IS device, delivered using standardized protocols, for treating similar types of surgeries.

Of note, some limitations should be underscored. In addition, there is a need for clear and complete reporting of outcome data for the interventions being compared. All trials included in this review had important methodological limitations. Although blinding of participants and personnel may be very difficult from a practical perspective, several other factors such as the lack of blinding of outcome assessors, loss to follow-up, and the absence of intention-to-treat analyses were common methodological limitations in the available studies.

Overall, due to the serious risk of bias and imprecision, the overall certainty of the available evidence is very low, and several questions persist. Thus, it is unclear whether IS used alone or in combination with other therapies is effective when compared to other interventions used alone or in combination.

Moreover, although some studies concluded that IS was safe, the available information on adverse events was insufficient to perform a comprehensive meta-analysis that could provide more accurate results on the safety of IS. The evidence is currently insufficient to support or refute the routine use of IS after cardiac surgeries. The results of the six ongoing RCTs are necessary to provide more precise and reliable information on which to base further trials and protocols, and to guide clinical decision-making processes on the use of IS after cardiac surgeries.

We believe the strengths of this systematic review include transparency, rigid methods, assessment of the quality of evidence for each outcome, and extensive and careful searches, with no restrictions on language or publication date. We searched the gray literature database and ongoing studies and performed a rigorous critical assessment of the current body of evidence. Furthermore, the assessment of certainty of evidence using the GRADE approach is paramount in pointing out limitations in current trials and upon which to base further high quality RCTs. Another strong point of this review was the separate analysis by the type of IS (flowor volume-oriented IS), when possible. This high-quality review underlines that there is an urgent need to conduct high-quality RCTs in this field.

### Limitations

We consider as limitations of this systematic review the inclusion of biased clinical trials, such as those with lack of blinding of outcome assessors, or without adequate randomization; substantial heterogeneity among studies that made them unsuitable for meta-analysis; or studies with small samples that do not allow us to provide accurate estimates of the effects. As another limitation, we were unable to explain the heterogeneity in the meta-analysis of the PaO_2_ and SO_2_ outcomes.

## CONCLUSION

This meta-analysis revealed that IS was not superior to standard respiratory care for PPC and clinical outcomes, therefore its use should not be widely recommended until high-quality further studies are performed to ensure this clinical guidance.

## Supplement 1 Search strategy

**Table:**

14.08.2020

Medical Literature Analysis and Retrieval System Online (or MEDLINE®) via PubMed® - 175 results

#1 ("Cardiac Surgical Procedures"[Mesh]) OR "Heart Surg^*^" OR "Cardiac Surg^*^" OR "Cardiovascular Surg^*^" OR ("Coronary Artery Bypass"[Mesh]) OR (Coronary Artery Bypass Grafting) OR CABG OR (Heart Bypass) OR (Coronary Bypass) OR (Aortocoronary Bypass) OR ("Myocardial Revascularization"[Mesh]) OR (Transmyocardial Revascularization) OR (Heart Myectomy) OR (Heart Myotomy) OR ("Cardiopulmonary Bypass"[Mesh]) OR (Heart-Lung Bypass) OR (Cardiology Robotic Surgery) OR ("Angioplasty"[Mesh]) OR ("Balloon Valvuloplasty"[Mesh]) OR (Valve Repair) OR (Valvular Surgery) OR (Valve Surgery) OR ("Cardiac Valve Annuloplasty"[Mesh]) OR Annuloplasty OR (Cardiac Valve Annulus Repair) OR (Heart Valve Annulus Repair) OR (Cardiac Valve Annular Reduction) OR (Cardiac Valve Annulus Shortening) OR (Cardiac Valve Annulus Reduction) OR (Valve Replacement) OR ("Transcatheter Aortic Valve Replacement"[Mesh]) OR TAVR OR ("Heart Valve Prosthesis Implantation"[Mesh]) OR (Insertion of Pacemaker) OR (Insertion of implantable cardioverter defibrillator) OR (Maze Surgery) OR (Aortic Aneurysm Repair) OR (Aortic Surgery) OR ("Heart Transplantation"[Mesh]) OR "Heart Transplant^*^" OR (Heart Grafting) OR "Cardiac Transplant^*^" OR (Insertion of Ventricular Assist Device) OR (VAD Surgery) OR (Insertion of Total Artificial Heart) OR ("Thoracic Surgery"[Mesh]) OR ("Thoracic Surgical Procedures"[Mesh]) OR "Thoracic Surg^*^" OR (Arrhythmia Surgery) OR (Left Ventricular Remodeling) OR (Surgical Ventricular Restoration) OR (Atrial Fibrillation Ablation) OR (Atrial Fibrillation Surgery) OR (Hypertrophic Cardiomyopathy Surgery) OR (Thoracoscopic Surgical Procedures) OR (Thoracoscopic Surgeries) OR ("Thoracotomy"[Mesh]) OR Thoracotomies OR Thoracostomy OR ("Thoracic Surgery, Video-Assisted"[Mesh]) OR (Video-Assisted Thoracic Surgery) OR VATS

#2 ("Breathing Exercises"[Mesh]) OR "Incentive Spiromet^*^" OR (Flow-Incentive Spirometer) OR Triflo OR Triflow OR Voldyne OR respiron

#3 ((((clinical[Title/Abstract] AND trial[Title/Abstract]) OR clinical trials as topic[MeSH Terms] OR clinical trial[Publication Type] OR random^*^[Title/Abstract] OR random allocation[MeSH Terms] OR therapeutic use[MeSH Subheading])))

#4 #1 AND #2 AND #3

Embase® via Elsevier - 304 results #1 'heart surgery'/exp OR 'heart surg^*^' OR 'cardiac surg^*^' OR 'cardiosurgery' OR 'heart operation' OR 'myocardial resection' OR 'surgery, heart' OR 'open heart surgery'/exp OR 'intracardiac surgery' OR 'minimally invasive cardiac surgery'/exp OR 'coronary artery bypass graft'/exp OR 'coronary artery bypass' OR 'aorta coronary artery bypass' OR 'aorta coronary bypass' OR 'aorta coronary vein bypass' OR 'aorta coronary vein shunt' OR 'aortic coronary artery bypass' OR 'aortic coronary bypass' OR 'aorticocoronary anastomosis' OR 'aorto coronary artery bypass' OR 'aorto coronary bypass' OR 'aorto coronary vein bypass' OR 'aortocoronary anastomosis' OR 'aortocoronary artery bypass' OR 'aortocoronary artery bypass' OR 'aortocoronary bypass' OR 'aortocoronary shunt' OR 'aortocoronary vein bypass' OR 'aortocoronary venous bypass' OR 'coronary artery graft' OR 'coronary bypass' OR 'coronary vein bypass' OR 'coronary venous bypass' OR 'heart muscle revascularization'/exp OR 'heart muscle revascularization' OR 'heart muscle revascularisation' OR 'anastomosis, internal mammary artery' OR 'artery implantation, mammary' OR 'implantation, internal mammary artery' OR 'internal mammary arterial anastomosis' OR 'internal mammary arterial implantation' OR 'internal mammary artery anastomosis' OR 'internal mammary artery graft' OR 'internal mammary artery implant' OR 'internal mammary artery implantation' OR 'internal mammary artery reimplantation' OR 'internal mammary-coronary artery anastomosis' OR 'mammary arterial implantation' OR 'mammary artery implantation' OR 'cardiac muscle revascularisation' OR 'cardiac muscle revascularization' OR 'myocardium revascularization' OR 'myocardium revascularisation' OR 'coronary revascularisation' OR 'coronary revascularization' OR 'heart revascularisation' OR 'heart revascularization' OR 'myocardial revascularisation' OR 'myocardial revascularization' OR 'revascularisation, transmyocardial laser' OR 'revascularization, transmyocardial laser' OR 'transmyocardial laser revascularisation' OR 'transmyocardial laser revascularization' OR 'vineberg operation' OR 'cardiopulmonary bypass'/exp OR 'cardiopulmonary bypass' OR 'atriopulmonary shunt' OR 'bypass, cardiopulmonary' OR 'cardiopulmonary shunt' OR 'heart lung bypass' OR 'angioplasty'/exp OR 'angioplasty' OR 'transluminal coronary angioplasty'/exp OR 'coronary artery dilatation, transluminal' OR p.t.c.a. OR ptca OR 'transluminal valvuloplasty'/exp OR 'transluminal valvuloplasty' OR 'balloon valvotomy' OR valvuloplasty OR valvulotomy OR 'valve repair' OR valvotomy OR valvulotomy OR 'annuloplasty'/exp OR annuloplasty OR 'heart valve replacement'/exp OR 'valve replacement' OR 'valvular replacement' OR 'valve implantation' OR 'valve prosthesis implantation' OR 'valvular replacement' OR 'transcatheter aortic valve implantation'/exp OR TAVI OR 'pacemaker implantation'/exp OR 'pacemaker implantation' OR 'artificial heart pacemaker implantation' OR 'heart pacemaker implantation' OR 'maze procedure'/exp OR 'maze procedure' OR 'Cox maze operation' OR 'Cox maze procedure' OR 'Cox maze surgery' OR 'Cox-maze ablation' OR 'Cox-maze technique' OR 'maze ablation' OR 'maze operation' OR
'maze surgery' OR 'maze technique' OR 'surgical Cox-maze procedure' OR 'surgical maze' OR 'aortic aneurysm surgery'/exp OR 'aortic aneurysm surgery' OR 'aortic surgery'/exp OR 'aortic surgery' OR 'aorta surgery' OR 'aortopexy' OR 'surgery, aorta' OR 'heart transplantation'/exp OR 'heart transplant^*^' OR 'cardiac transplant^*^' OR 'heart allograft' OR 'heart allotransplantation' OR 'heart heterograft' OR 'transplant, heart' OR 'heart heterotransplantation' OR 'heart homograft' OR 'heart homotransplantation' OR 'heart orthotopic transplantation' OR 'heart tissue transplantation' OR 'heart ventricle transplantation' OR 'heart graft'/exp OR 'heart graft' OR 'cardiac graft' OR 'graft, heart' OR 'Ventricular Assist Device Surg^*^' OR 'Insertion of Total Artificial Heart' OR 'thorax surgery'/exp OR 'thorax surg^*^' OR 'cardiothoracic surg^*^' OR 'chest surg^*^' OR 'chest wall surg^*^' OR 'surgery, chest' OR 'surgery, thoracic' OR 'surgery, thorax' OR 'thoracic operation' OR 'thoracic surg^*^' OR 'left ventricular remodeling'/exp OR 'left ventricular remodeling' OR 'surgical ventricular restoration'/exp OR 'surgical ventricular restoration' OR 'atrial fibrillation ablation'/exp OR 'atrial fibrillation ablation' OR 'thoracoscopic surgery'/exp OR 'thoracoscopic surg^*^' OR 'thoracotomy'/exp OR thoracotomy OR 'video assisted thoracoscopic surgery'/exp OR 'video assisted thoracoscopic surg^*^'
#2 'breathing exercise'/exp OR 'breathing exercise' OR 'incentive spirometry'/exp OR 'incentive spiromet^*^' OR 'incentive spirometer'/exp OR 'Respiflo 5000' OR Coach OR Triflo OR Triflow OR Voldyne OR respiron

#3 'crossover procedure':de OR 'double-blind procedure':de OR 'randomized controlled trial':de OR 'single-blind procedure':de OR (random^*^ OR factorial^*^ OR crossover^*^ OR cross NEXT/1 over^*^ OR placebo^*^ OR doubl^*^ NEAR/1 blind^*^ OR singl^*^ NEAR/1 blind^*^ OR assign^*^ OR allocat^*^ OR volunteer^*^):de,ab,ti

#4 #1 AND #2 AND #3

Cochrane Central Register of Controlled Trials (or CENTRAL) via Cochrane Library - 136 results
#1 MeSH descriptor: [Cardiac Surgical Procedures] explode all trees
#2 MeSH descriptor: [Coronary Artery Bypass] explode all trees
#3 MeSH descriptor: [Myocardial Revascularization] explode all trees
#4 MeSH descriptor: [Cardiopulmonary Bypass] explode all trees
#5 MeSH descriptor: [Angioplasty] explode all trees
#6 MeSH descriptor: [Balloon Valvuloplasty] explode all trees
#7 MeSH descriptor: [Cardiac Valve Annuloplasty] explode all trees
#8 MeSH descriptor: [Transcatheter Aortic Valve Replacement] explode all trees
#9 MeSH descriptor: [Heart Valve Prosthesis Implantation] explode all trees
#10 MeSH descriptor: [Heart Transplantation] explode all trees
#11 MeSH descriptor: [Thoracic Surgical Procedures] explode all trees
#12 MeSH descriptor: [Thoracotomy] explode all trees
#13 MeSH descriptor: [Thoracic Surgery, Video-Assisted] explode all trees
#14 MeSH descriptor: [Thoracic Surgery] explode all trees
#15 "Heart Surg^*^" OR "Cardiac Surg^*^" OR "Cardiovascular Surg^*^" OR (Coronary Artery Bypass Grafting) OR CABG OR (Heart Bypass) OR (Coronary Bypass) OR (Aortocoronary Bypass) OR (Myocardial Revascularization) OR (Transmyocardial Revascularization) OR (Heart Myectomy) OR (Heart Myotomy) OR (Heart-Lung Bypass) OR (Cardiology Robotic Surgery) OR (Valve Repair) OR (Valvular Surgery) OR (Valve Surgery) OR Annuloplasty OR (Cardiac Valve Annulus Repair) OR (Heart Valve Annulus Repair) OR (Cardiac Valve Annular Reduction) OR (Cardiac Valve Annulus Shortening) OR (Cardiac Valve Annulus Reduction) OR (Valve Replacement) OR TAVR OR (Insertion of Pacemaker) OR (Insertion of implantable cardioverter defibrillator) OR (Maze Surgery) OR (Aortic Aneurysm Repair) OR (Aortic Surgery) OR "Heart Transplant^*^" OR (Heart Grafting) OR "Cardiac Transplant^*^" OR (Insertion of Ventricular Assist Device) OR (VAD Surgery) OR (Insertion of Total Artificial Heart) OR "Thoracic Surg^*^" OR (Arrhythmia Surgery) OR (Left Ventricular Remodeling) OR (Surgical Ventricular Restoration) OR (Atrial Fibrillation Ablation) OR (Atrial Fibrillation Surgery) OR (Hypertrophic Cardiomyopathy Surgery) OR (Thoracoscopic Surgical Procedures) OR (Thoracoscopic Surgeries) OR Thoracotomies OR Thoracostomy OR (Video-Assisted Thoracic Surgery) OR VATS
#16 #1 OR #2 OR #3 OR #4 OR #5 OR #6 OR #7 OR #8 OR #9 OR #10 OR #11 OR #12 OR #13 OR #14 OR #15
#17 MeSH descriptor: [Breathing Exercises] explode all trees
#18 Incentive Spirometr^*^ OR (Flow-Incentive Spirometer) OR Coach OR Triflo OR Triflow OR Voldyne OR respiron
#19 #17 OR #18
#20 #16 AND #19
Latin American and Caribbean Health Sciences Literature (LILACS) via Virtual Health Library Regional Portal - 46 results
#1 MH:"Procedimentos Cirúrgicos Cardíacos" OR (Cardiac Surgical Procedures) OR (Procedimientos Quirúrgicos Cardíacos) OR MH:E04.100.376$ OR MH:E04.928.220$ OR MH:"Ponte de Artéria Coronária" OR (Coronary Artery Bypass) OR (Puente de Arteria Coronaria) OR (Derivação da Artéria Coronária) OR (Ponte Aortocoronária) OR MH:E04.100.376.719.332$ OR MH:E04.100.814.868.750$ OR MH:E04.928.220.520.220$ OR MH:"Ponte Cardiopulmonar" OR (Cardiopulmonary Bypass) OR (Puente Cardiopulmonar) OR (Circuito Cardiopulmonar) OR (Derivação Cardiopulmonar) OR (Ponte Coração-Pulmão) OR MH:E04.292.413$ OR MH:Angioplastia OR Angioplasty OR MH:E02.148.050 OR MH:E04.100.814.529.124$ OR MH:E04.502.382.124$ OR MH:E05.157.016$ OR MH:"Valvuloplastia com Balão" OR Valvuloplasty OR Valvuloplastia OR MH:E02.148.108$ OR MH:E05.157.125$ OR MH:"Anuloplastia da Valva Cardíaca" OR (Cardiac Valve Annuloplasty) OR (Anuloplastia de la Válvula Cardíaca) OR (Anuloplastia Valvar Cardíaca) OR (Anuloplastia da Válvula Cardíaca) OR MH:E04.100.376.062$ OR MH:E04.928.220.109$ OR MH:"Substituição da Valva Aórtica Transcateter" OR (Transcatheter Aortic Valve Replacement) OR (Reemplazo de la Válvula Aórtica Transcatéter) OR MH:E04.100.376.485.500$ OR MH:E04.650.410.500$ OR MH:E04.928.220.410.500$ OR MH:"Implante de Prótese de Valva Cardíaca" OR (Heart Valve Prosthesis Implantation) OR (Implantación de Prótesis de Válvulas Cardíacas) OR (Implantação de Prótese Valvar Cardíaca) OR (Implantação de Prótese de Valva) OR (Implantação de Prótese de Valva Cardíaca) OR (Implante de Prótese Valvar Cardíaca) OR (Implante de Prótese de Valva) OR MH:E04.100.376.485$ OR MH:E04.650.410$ OR MH:E04.928.220.410$ OR MH:"Transplante de Coração" OR (Heart Transplantation) OR (Trasplante de Corazón) OR (Enxerto Cardíaco) OR (Enxerto de Coração) OR (Transplantação Cardíaco) OR (Transplantação de Coração) OR (Transplante Cardíaco) OR MH:E04.100.376.475$ OR MH:E04.928.220.390$ OR MH:E04.936.450.475$ OR MH:"Procedimentos Cirúrgicos Torácicos" OR (Thoracic Surgical Procedures) OR (Procedimientos Quirúrgicos Torácicos) MH:E04.928$ OR MH:Toracotomia OR Thoracotomy OR Toracotomía OR MH:E04.928.760$ OR MH:"Cirurgia Torácica Vídeoassistida" OR MH:"Thoracic Surgery, Video-Assisted" OR (Cirugía Torácica Asistida por Video) OR CTVA OR VATS OR MH:E01.370.388.250.840.830$ OR MH:E01.370.388.250.950.830$ OR MH:E04.502.250.840.830$ OR MH:E04.502.250.950.830$ OR MH:E04.928.752.830$

#2 MH:"Exercícios Respiratórios" OR (Breathing Exercises) OR (Ejercicios Respiratorios) OR (Exercício Respiratório) OR (Exercícios para os Músculos Respiratórios) OR MH:E02.190.525.186$ OR MH:E02.779.474.124$ OR (Espirometria de incentivo) OR Voldyne OR Triflo OR Triflow

Filter: LILACS

PEDro - 1^st^ search - 47 results / 2^nd^ search - 81 results
#1 Heart Surg^*^ / Subdiscipline: Cardiothoracics / Therapy: Respiratory Therapy / Method: Clinical trial
#2 Cardiac Surg^*^ / Subdiscipline: Cardiothoracics / Therapy: Respiratory Therapy / Method: Clinical trial

Cumulative Index of Nursing and Allied Health (or CINAHL®) via EBSCO - 80 results
S1 (MM "Surgery, Cardiovascular+") OR (MM "Thoracic Surgery, Video-Assisted") OR (MM "Thoracic Surgery+") OR (MM "Coronary Artery Bypass+") OR (MM "Myocardial Revascularization+") OR (MM "Angioplasty, Transluminal, Percutaneous Coronary") OR (MM "Cardiopulmonary Bypass") OR (MM "Angioplasty+") OR (MM "Angioplasty, Balloon, Laser-Assisted") OR (MM "Angioplasty, Balloon+") OR (MM "Angioplasty, Laser+") OR (MM "Percutaneous Coronary Intervention") OR (MM "Balloon Dilatation+") OR (MM "Cardiac Valve Annuloplasty+") OR (MM "Mitral Valve Annuloplasty") OR (MM "Transcatheter Aortic Valve Implantation") OR (MM "Heart Valve Prosthesis") OR (MM "Heart Transplantation+") OR (MM "Heart-Lung Transplantation") OR (MM "Thoracotomy") OR (MM "Thoracostomy+")

S2 "Heart Surg^*^" OR "Cardiac Surg^*^" OR "Cardiovascular Surg^*^" OR (Coronary Artery Bypass Grafting) OR CABG OR (Heart Bypass) OR (Coronary Bypass) OR (Aortocoronary Bypass) OR (Myocardial Revascularization) OR (Transmyocardial Revascularization) OR (Heart Myectomy) OR (Heart Myotomy) OR (Heart-Lung Bypass) OR (Cardiology Robotic Surgery) OR (Valve Repair) OR (Valvular Surgery) OR (Valve Surgery) OR Annuloplasty OR (Cardiac Valve Annulus Repair) OR (Heart Valve Annulus Repair) OR (Cardiac Valve Annular Reduction) OR (Cardiac Valve Annulus Shortening) OR (Cardiac Valve Annulus Reduction) OR (Valve Replacement) OR TAVR OR (Insertion of Pacemaker) OR (Insertion of implantable cardioverter defibrillator) OR (Maze Surgery) OR (Aortic Aneurysm Repair) OR (Aortic Surgery) OR "Heart Transplant^*^" OR (Heart Grafting) OR "Cardiac Transplant^*^" OR (Insertion of Ventricular Assist Device) OR (VAD Surgery) OR (Insertion of Total Artificial Heart) OR "Thoracic Surg^*^" OR (Arrhythmia Surgery) OR (Left Ventricular Remodeling) OR (Surgical Ventricular Restoration) OR (Atrial Fibrillation Ablation) OR (Atrial Fibrillation Surgery) OR (Hypertrophic Cardiomyopathy Surgery) OR (Thoracoscopic Surgical Procedures) OR (Thoracoscopic Surgeries) OR Thoracotomies OR Thoracostomy OR (Video-Assisted Thoracic Surgery) OR VATS S3 S1 OR S2
S4 (MM "Breathing Exercises+")

S5 "Incentive Spiromet^*^" OR (Flow-Incentive Spirometer) OR Triflo OR Triflow OR Voldyne OR respiron

S6 S4 OR S5

S7 S3 AND S6

S8 TX allocat^*^ random^*^ OR (MH "Quantitative Studies") OR (MH "Placebos") OR TX placebo^*^ OR TX random^*^ allocat^*^ OR (MH "Random Assignment") OR TX randomi^*^ control^*^ trial^*^ OR TX ((singl^*^ n1 blind^*^) OR (singl^*^ n1 mask^*^) ) OR TX ( (doubl^*^ n1 blind^*^) OR (doubl^*^ n1 mask^*^) ) OR TX ( (tripl^*^ n1 blind^*^) OR (tripl^*^ n1 mask^*^) ) OR TX ( (trebl^*^ n1 blind^*^) OR (trebl^*^ n1 mask^*^) ) OR TX clinic^*^ n1 trial^*^ OR PT Clinical trial OR (MH "Clinical Trials+")

S9 S7 AND S9

Scopus® via Elsevier - 810 results
#1 TITLE-ABS-KEY("Cardiac Surgi^*^" OR "Coronary Artery Bypass" "Myocardial Revascularization" OR "Cardiopulmonary Bypass" OR Angioplasty "Balloon Valvuloplasty" OR "Cardiac Valve Annuloplasty" OR "Transcatheter Aortic Valve Replacement" "Heart Valve Prosthesis Implantation" OR "Heart Transplant^*^" OR "Thoracic Surg^*^")
#2 "Heart Surg^*^" OR "Cardiac Surg^*^" OR "Cardiovascular Surg^*^" OR "Coronary Artery Bypass Graf^*^" OR CABG OR "Heart Bypass" OR "Coronary Bypass" OR "Aortocoronary Bypass" OR "Myocardial Revascularization" OR "Transmyocardial Revascularization" OR "Heart Myectomy" OR "Heart Myotomy" OR "Heart-Lung Bypass" OR "Cardiology Robotic Surg^*^" OR "Valve Repair" OR "Valvular Surg^*^" OR "Valve Surg^*^" OR Annuloplasty OR "Cardiac Valve Annulus Repair" OR "Heart Valve Annulus Repair" OR "Cardiac Valve Annular Reduction" OR "Cardiac Valve Annulus Shortening" OR "Cardiac Valve Annulus Reduction" OR "Valve Replacement" OR TAVR OR "Insertion of Pacemaker" OR "Insertion of implantable cardioverter defibrillator" OR "Maze Surg^*^" OR "Aortic Aneurysm Repair" OR "Aortic Surg^*^" OR "Heart Transplant^*^" OR "Heart Graft^*^" OR "Cardiac Transplant^*^" OR "Insertion of Ventricular Assist Device" OR "VAD Surg^*^" OR "Insertion of Total Artificial Heart" OR "Thoracic Surg^*^" OR "Arrhythmia Surg^*^" OR "Left Ventricular Remodeling" OR "Surgical Ventricular Restoration" OR "Atrial Fibrillation Ablation" OR "Atrial Fibrillation Surg^*^" OR "Hypertrophic Cardiomyopathy Surg^*^" OR "Thoracoscopic Surg^*^" OR Thoracotom^*^ OR Thoracostomy OR "Video-Assisted Thoracic Surg^*^" OR VATS
#3 #1 OR #2
#4 TITLE-ABS-KEY("Breathing Exercis^*^")
#5 "Incentive Spirometr^*^" OR (Flow-Incentive Spirometer) OR Coach OR Triflo OR Triflow OR Voldyne OR respiron
#6 #4 OR #5
#7 #3 AND #6
#8 TITLE-ABS-KEY((clinic^*^ w/1 trial^*^) OR (randomi^*^ w/1 control^*^) OR (randomi^*^ w/2 trial^*^) OR (random^*^ w/1 assign^*^) OR (random^*^ w/1 allocat^*^) OR (control^*^ w/1 clinic^*^) OR (control^*^ w/1 trial) OR placebo^*^ OR (Quantitat^*^ w/1 Stud^*^) OR (control^*^ w/1 stud^*^) OR (randomi^*^ w/1 stud^*^) OR (singl^*^ w/1 blind^*^) OR (singl^*^ w/1 mask^*^) OR (doubl^*^ w/1 blind^*^) OR (doubl^*^ w/1 mask^*^) OR (tripl^*^ w/1 blind^*^) OR (tripl^*^ w/1 mask^*^) OR (trebl^*^ w/1 blind^*^) OR (trebl^*^ w/1 mask^*^)) AND NOT (SRCTYPE(b) OR SRCTYPE(k) OR SRCTYPE(p) OR SRCTYPE(r) OR SRCTYPE(d) OR DOCTYPE(ab) OR DOCTYPE(bk) OR DOCTYPE(ch) OR DOCTYPE(bz) OR DOCTYPE(cr) OR DOCTYPE(ed) OR DOCTYPE(er) OR DOCTYPE(le) OR DOCTYPE(no) OR DOCTYPE(pr) OR DOCTYPE(rp) OR DOCTYPE(re) OR DOCTYPE(sh))
#9 #7 AND #8

SciELO - 1^st^ search - 3 results / 2^nd^ search - 4 results
#1 (Heart Surg^*^) AND (Incentive Spiromet^*^)
#2 (Cardiac Surg^*^) AND (Incentive Spiromet^*^)

OpenGrey Database - 0 results
Incentive Spiromet^*^
ClinicalTrials.gov - 6 results
Condition: (Cardiac Surg^*^) OR (Heart Surg^*^) OR (Thoracic Surg^*^)
Other terms: (Breathing Exercis^*^) OR (Incentive Spiromet^*^) OR (Flow-Incentive Spirometer) OR Triflo OR Triflow OR Voldyne OR respiron

clinicaltrialsregister.eu - 0 results
#1 Incentive Spiromet^*^

Rebec - 0 results
#1 Cardiac Surg^*^
#2 Heart Surg^*^

World Health Organization International Clinical Trials Registry Platform - No access at the time of search.
#1 (Heart Surg^*^) AND (Incentive Spiromet^*^)
#2 (Cardiac Surg^*^) AND (Incentive Spiromet^*^)

## Appendix

**Supplement 2:** Assessment of certainty of evidence.

Question 1: Incentive spirometry *vs.* respiratory care in patients undergoing cardiac surgery.												
Question 2: Incentive spirometry *vs.* other therapies in patients undergoing cardiac surgery.												
Question 3: Incentive spirometry *vs.* noninvasive ventilation in patients undergoing cardiac surgery.												

Certainty assessment							No. of patients		Effect		Certainty	Importance
No. of studies	Study design	Risk of bias	Inconsistency	Indirectness	Imprecision	Other considerations	Incentive spirometry	Control	Relative (95% CI)	Absolute (95% CI)		
IS *vs.* Respiratory care - Postoperative pulmonary complications (assessed with: number of events [pneumonia and atelectasis] recorded)												
4	Randomized trials	^*^Very serious^a,b,c,d^	Not serious	Not serious	Not serious	None	45/189 (24.2%)	65/203 (32.0%)	RR 0.91 (0.72 to 1.14)	29 fewer per 1,000 (from 90 fewer to 45 more)	⨁⨁◯◯ Low	Critical
IS *vs.* Respiratory care - LOS (assessed with: number of days spent in hospital)												
3	Randomized trials	^*^Very serious^a,b,c,d,j^	Not serious	Not serious	^*^Serious^i^	None	145	146	-	MD 1.38 lower (2.96 lower to 0.21 higher)	⨁◯◯◯ Very low	Important
IS *vs.* Respiratory care - Length of ICU stay (assessed with: number of days spend in ICU)												
2	Randomized trials	^*^Very serious^b,c,e,f,g,h^	Not serious	Not serious	^*^Serious^i^	None	26	26	-	MD 0.78 lower (1.61 lower to 0.06 higher)	⨁◯◯◯ Very low	Critical
IS *vs.* Respiratory care - Peak of expiratory flow (assessed with: spirometry [L/min])												
2	Randomized trials	^*^Very s erious^b,c,e,f^	Not serious	Not serious	^*^Serious^i^	None	49	65	-	MD 0.59 lower (1.97 lower to 0.78 higher)	⨁◯◯◯ Very low	Important
IS *vs.* Respiratory care - Forced expiratory volume in one second (assessed with: spirometry [% and liters])												
5	Randomized trials	^*^Very serious^b,c,e,f^	Not serious	Not serious	Not serious	None	203	200	-	SMD 0.16 SD lower (0.48 lower to 0.16 higher)	⨁⨁◯◯ Low	Important
IS *vs.* Respiratory care - Forced vital capacity (assessed with: spirometry [% and liters])												
5	Randomized trials	^*^Very serious^b,c,e,f^	^*^Serious^k^	Not serious	Not serious	None	203	200	-	SMD 0.14 SD higher (0.4 lower to 0.67 higher)	⨁◯◯◯ Very low	Important
IS *vs.* Respiratory care - Vital capacity (assessed with: spirometry [% and liters])												
3	Randomized trials	^*^Very serious^b,c,e,f,j,l^	^*^Serious^k^	Not serious	Not serious	None	57	73	-	SMD 0.38 SD higher (0.59 lower to 1.34 higher)	⨁◯◯◯ Very low	Important
IS *vs.* Respiratory care - Arterial oxygen partial pressure (mmHg)												
6	Randomized trials	^*^Very serious^b,c,e,f,j,l^	^*^Serious^k^	Not serious	Not serious	None	125	157	-	MD 4.48 lower (8.32 lower to 0.63 lower)	⨁◯◯◯ Very low	Critical
IS *vs.* Respiratory care - Oxygen saturation (%)												
3	Randomized trials	^*^Very serious^b,c,e,f,j,l^	^*^Serious^k^	Not serious	Not serious	None	71	71	-	MD 2.54 higher (1.74 lower to 6.82 higher)	⨁◯◯◯ Very low	Critical
IS *vs.* Other therapies - Postoperative pulmonary complications (assessed with: number of events [pneumonia and atelectasis] recorded)												
3	Randomized trials	^*^Very serious^b,c,e,f,l^	^*^Serious^k^	Not serious	Not serious	None	38/54 (70.4%)	63/95 (66.3%)	RR 1.04 (0.73 to 1.49)	27 more per 1,000 (from 179 fewer to 325 more)	⨁◯◯◯ Very low	Critical
IS *vs.* Other therapies - Forced expiratory volume in one second (assessed with: spirometry [liters])												
2	Randomized trials	^*^Very serious^b,c,e,f^	Not serious	Not serious	^*^Serious^i^	None	25	50	-	MD 0.08 higher (0.08 lower to 0.25 higher)	⨁◯◯◯ Very low	Important
IS *vs.* Other therapies - Forced vital capacity (assessed with: spirometry [% and liters])												
3	Randomized trials	^*^Very serious^b,c,e,f,j,l^	Not serious	Not serious	^*^Serious^i^	None	37	73	-	SMD 0.15 SD higher (0.25 lower to 0.55 higher)	⨁◯◯◯ Very low	Important
IS *vs.* Other therapies - Arterial oxygen partial pressure (mmHg)												
3	Randomized trials	^*^Very serious^b,c,e,f,j,l^	^*^Serious^k^	Not serious	Not serious	None	44	87	-	MD 3.63 lower (9.18 lower to 1.93 higher)	⨁◯◯◯ Very low	Critical
IS *vs.* NIV - Postoperative pulmonary complications (assessed with: number of events [pneumonia and atelectasis] recorded)												
4	Randomized trials	^*^Very serious^b,c,e,f,l^	^*^Serious^k^	Not serious	Not serious	None	95/113 (84.1%)	106/138 (76.8%)	"RR 1.14 (0.84 to 1.55)"	108 more per 1,000 (from 123 fewer to 422 more)	⨁◯◯◯ Very low	Critical
IS *vs.* NIV - Mortality (assessed with: number of events recorded)												
2	Randomized trials	^*^Very serious^b,c,e,f,h^	Not serious	Not serious	^*^Serious^i^	None	0/65 (0.0%)	4/114 (3.5%)	RR 0.49 (0.08 to 2.93)	18 fewer per 1,000 (from 32 fewer to 68 more)	⨁◯◯◯ Very low	Critical
IS *vs.* NIV - Adverse events (assessed with: number of events recorded)												
3	Randomized trials	^*^Very serious^b,c,e,f,l^	Not serious	Not serious	^*^Serious^i^	None	13/61 (21.3%)	14/81 (17.3%)	RR 1.10 (0.62 to 1.95)	17 more per 1,000 (from 66 fewer to 164 more)	⨁◯◯◯ Very low	Critical
IS *vs.* NIV - Arterial oxygen partial pressure (mmHg)												
4	Randomized trials	^*^Very serious^b,c,e,f,l^	^*^Serious^k^	Not serious	Not serious	None	96	120	-	MD 2.95 higher (4.69 lower to 10.58 higher)	⨁◯◯◯ Very low	Critical
IS *vs.* NIV - Oxygen saturation (%)												
2	Randomized trials	^*^Very serious^b,c,e,f,h^	^*^Serious^k^	Not serious	Not serious	None	56	92	-	MD 0.99 lower (2.12 lower to 0.14 higher)	⨁◯◯◯ Very low	Critical

CI=confidence interval; ICU=intensive care unit; IS=incentive spirometry; LOS=length of stay; MD=mean difference; NIV=noninvasive ventilation; RR=risk ratio; SD=standard deviation; SMD=standardized mean difference

^*^For very serious limitations, we downgraded two levels, and for serious limitations, we downgraded one level

^a^. Allocation was not concealed in most studies

^b^. There was no blinding of all subjects in the studies

^c^. There was no blinding of all therapists who administered the therapy

^d^. Data for at least one key outcome was not analyzed by “intention to treat” in most studies

^e^. Allocation was not concealed

^f^. Data for at least one key outcome was not analyzed by “intention to treat”

^g^. Measures of at least one key outcome were not obtained from > 85% of the subjects initially allocated to groups

^h^. There was no blinding of all assessors who measured at least one key outcome

^i^. Imprecision with few studies and few participants

^j^. Measures of at least one key outcome were not obtained from > 85% of the subjects initially allocated to groups in most studies

^l^. There was no blinding of all assessors who measured at least one key outcome in most studies
